# Hypoxia rescues complex 1-associated disease caused by proteostatic defects

**DOI:** 10.1038/s42255-026-01566-0

**Published:** 2026-07-08

**Authors:** Ankur Garg, Brandon R. Desousa, Raju Roy, Amy Flis, Skyler Y. Blume, Yohei Abe, Arthur A. Melo, Ryan R. Cupo, Gabriela Grigorean, Daniel R. Southworth, James Shorter, Isha H. Jain

**Affiliations:** 1 Gladstone Institutes, San Francisco, CA USA; 2 Arc Institute, Palo Alto, CA USA; 3 Biomedical Sciences Program, University of California San Francisco, San Francisco, CA USA; 4 Department of Biochemistry and Biophysics, Perelman School of Medicine, University of Pennsylvania, Philadelphia, PA USA; 5 Department of Biochemistry and Biophysics, University of California San Francisco, San Francisco, CA USA; 6 Biophysics Graduate Program, University of California San Francisco, San Francisco, CA USA; 7 Institute for Neurodegenerative Diseases, University of California San Francisco, San Francisco, CA USA; 8 MitoCare Center for Mitochondrial Imaging Research and Diagnostics, Department of Pathology and Genomic Medicine, Thomas Jefferson University, Philadelphia, USA; 9 Proteomics Core, Genome and Biomedical Sciences Facility, University of California Davis, Davis, CA USA

**Keywords:** Mitochondria, Protein aggregation, Energy metabolism, Metabolism, Proteases

## Abstract

Impaired mitochondrial proteostasis underlies a broad spectrum of diseases, yet effective therapies remain limited. Here we show that deficiency of HTRA2, a mitochondrial intermembrane space protease, can be rescued by hypoxia therapy. Using an *Htra2* mutant mouse model that displays severe neurodegeneration and early lethality, we find that continuous hypoxia rescues striatal degeneration and extends lifespan. Mechanistically, we demonstrate that HTRA2 forms a functional complex with the disaggregase CLPB. Loss of function of either protein drives aggregation of intermembrane space-facing subunits of complex I of the electron transport chain, resulting in secondary complex I dysfunction. These changes impair tissue oxygen consumption and probably cause pathological hyperoxia, which is corrected by hypoxia. Together, these findings define a proteostasis pathway linking intermembrane space quality control to complex I function and expand the potential of hypoxia therapy to secondary complex I disease.

## Abstract (web summary)

The HTRA2–CLPB proteostasis system is a mitochondrial quality control module whose dysfunction drives local oxygen toxicity, which can be mitigated by lowering ambient oxygen levels.

## Main

The mitochondrial electron transport chain (ETC) consumes over 90% of cellular oxygen to fuel aerobic ATP production and prevent toxic oxygen accumulation in tissues^1^. This dual role of oxygen—as both a substrate and a toxin—demands tight coordination between oxygen delivery and mitochondrial consumption. Among the five ETC complexes, complex I (C1) is the largest and most intricate. It is composed of over 40 subunits organized into functional modules for NADH oxidation (N-module), quinone binding (Q-module) and proton pumping (P-module). Electron flow through C1 depends on fragile iron–sulfur clusters (ISCs), which are especially susceptible to oxidative damage^2^. C1 dysfunction has been implicated in a wide spectrum of diseases—from rare inborn errors such as Leigh syndrome, caused by mutations in C1 subunits or assembly factors, to more common neurodegenerative conditions such as Parkinson’s disease^3–5^.

We previously demonstrated that hypoxia is a powerful therapeutic strategy for mitochondrial disease. In a mouse model of Leigh syndrome caused by loss of the C1 subunit Ndufs4, exposure to 11% oxygen extended survival and reversed neurodegeneration^6^. This effect was not mediated by canonical hypoxia-inducible factor (HIF) signalling but instead reflected a pathogenic accumulation of excess oxygen that was normalized by hypoxic breathing^7^. More recently, we conducted a genome-wide screen and identified over 70 additional disease genes whose loss-of-function could be rescued by hypoxia in vitro^8^. While these findings suggest broad therapeutic potential, they are limited to cell culture. It remains unclear whether hypoxia has similar efficacy across disease contexts in vivo and whether the mechanism of rescue is shared across genetically distinct disorders, beyond primary C1 lesions. Understanding the common pathophysiological features of these hypoxia-responsive diseases will help elucidate which ones are likely to benefit from hypoxia and why. In this study, we use the disease gene hit, *HTRA2*, to explore these key questions.

Mitochondrial proteostasis presents unique challenges due to high local production of reactive oxygen species and the absence of a proteasome within the organelle. Instead, there is a diverse array of dedicated proteases and chaperones that maintain protein quality control in this challenging protein folding microenvironment^9,10^. Approximately 20 resident mitochondrial proteases have been identified and play key roles in mitochondrial surveillance, turnover and stress responses. Among them, HTRA2 is a serine protease located in the mitochondrial intermembrane space (IMS). It is the mammalian homologue of the bacterial protein DegP, which functions as a chaperone at low temperatures and a protease at elevated temperatures^11^. HTRA2 is linked to a wide range of mitochondrial processes—including apoptosis, cristae morphology and mitochondrial dynamics^12–16^. Despite these associations, the precise biochemical consequences of HTRA2 deficiency that drive cellular dysfunction and disease remain incompletely understood.

**Fig. 1:**
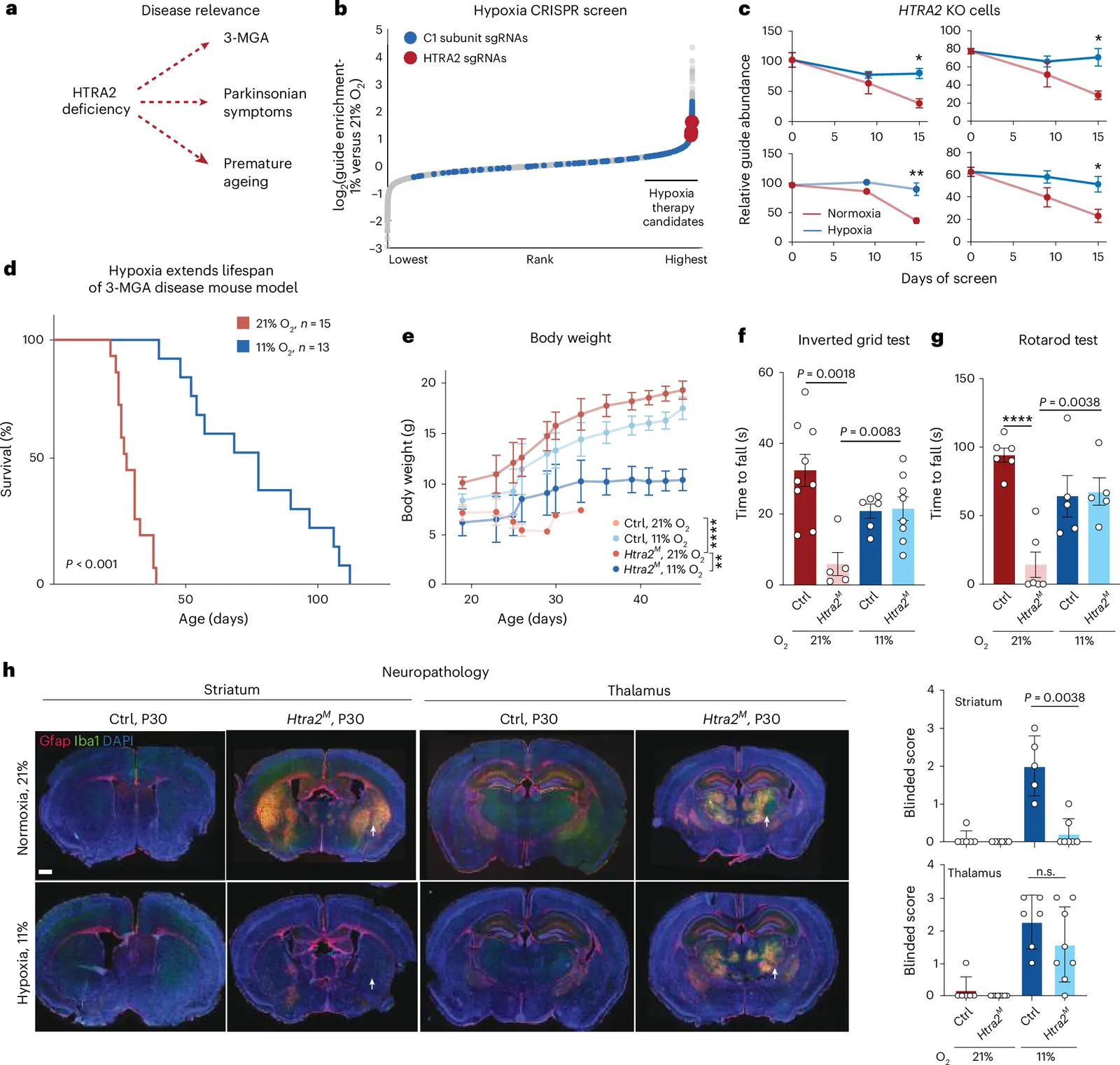
Hypoxia extends the lifespan of *Htra2* mutant mice. **a**, HTRA2 deficiency is associated with pathologies ranging from 3-MGA to Parkinson’s disease to premature ageing. **b**, A genome-wide CRISPR KO screen in K562 cells exposed to normoxia (21% O_2_) or hypoxia (1% O_2_) identifies *HTRA2* KO as a candidate for hypoxia therapy (reanalysis of supplemental data from ref. ^8^). All C1 subunit guide RNAs (gRNAs) are shown as blue dots. The four gRNAs targeting *HTRA2* are shown as red dots. **c**, Plots of the normalized abundance of the four *HTRA2*-targeting gRNAs over the duration of screen^8^. The gRNAs are depleted over time in normoxia suggesting a fitness defect in *HTRA2* KO cells. They are not depleted in hypoxia, suggesting that the fitness defect is rescued by hypoxia (*n* = 3 per collection day D0 and D15 timepoints, *n* = 2 for D9 time point). Data are presented as mean ± s.e.m. from two to three biological replicates. **d**–**g**, Kaplan–Meier survival curve (**d**), body weight (data are presented as mean ± s.d.) (**e**), time on inverted grid (data are presented as mean ± s.e.m.) (**f**) and time on rotarod (data are presented as mean ± s.e.m.) (**g**) of control (Ctrl) and *Htra2* mutant (*Htra2*^*M*^) mice exposed to continuous inhaled hypoxia or normoxia. Sample sizes and sex distribution for behavioural assays: survival—males, mutant 21% O_2_ (*n* = 6), 11% O_2_ (*n* = 6); females, mutant 21% O_2_ (*n* = 9), 11% O_2_ (*n* = 7); inverted grid—males, 21% O_2_ Ctrl (*n* = 6), mutant (*n* = 5); 11% O_2_ Ctrl (*n* = 4), mutant (*n* = 3); females, 21% O_2_ Ctrl (*n* = 3), mutant (*n* = 0); 11% O_2_ Ctrl (*n* = 2), mutant (*n* = 5); rotarod—males, 21% O_2_ Ctrl (n = 4), mutant (*n* = 3); 11% O_2_ Ctrl (*n* = 2), mutant (*n* = 2); females: 21% O_2_ Ctrl (*n* = 1), mutant (*n* = 2); 11% O_2_ Ctrl (*n* = 3), mutant (*n* = 3). **h**, Representative neuropathology (Iba1 and Gfap staining) of *Htra2*^*M*^ and control mice at P30 after chronic exposure to normoxia or hypoxia. Brain sections with striatum and thalamus regions are shown (left; white arrows, scale bar, 300 μm) and lesions quantified based on blind scoring (right). Data are presented as mean ± s.d. Sample sizes for neuropathology: striatum—Ctrl (21% O_2_, *n* = 6; 11% O_2_, *n* = 6), mutant (21% O_2_, *n* = 5; 11% O_2_, *n* = 7); thalamus—Ctrl and mutant (21% O_2_, *n* = 6 each; 11% O_2_, *n* = 8 each). For bar plots, *P* values were calculated by an unpaired two-tailed *t*-test; for survival assays, *P* values were calculated by log-rank (Mantel–Cox) tests. *P < 0.05, **P < 0.005, ***P < 0.0005, ****P < 0.0001, n.s., not significant. Source data

HTRA2 has been implicated in a range of diseases, spanning from rare inborn errors of metabolism to age-associated neurodegeneration (Fig. 1a). Complete loss-of-function mutations in *HTRA2* cause a severe childhood disorder known as 3-methylglutaconic aciduria (3-MGA), characterized by progressive neurodegeneration and early death^17,18^. While there are over a dozen genetic causes of 3-MGA, the underlying biochemical mechanisms remain poorly understood and no effective treatments are currently available. In addition to its role in rare disease, *HTRA2*, also known as *PARK13*, has been associated with familial Parkinson’s disease^19^. Although the pathogenicity and penetrance of *HTRA2* variants in humans remain under debate, a naturally occurring loss-of-function mutation in mice leads to Parkinsonian motor defects^20,21^. Moreover, HTRA2 phosphorylation is decreased in the brains of patients with Parkinson’s disease carrying *PINK1* mutations^22^ and HTRA2 has been shown to degrade oligomeric α-Syn^23^. Intriguingly, HtrA2 loss in non-neuronal cells is associated with a premature ageing phenotype in mice^24^. Given its genetic links to diverse disorders—from paediatric mitochondrial disease to Parkinson’s and ageing—we chose to investigate *HTRA2* as a model for understanding mitochondrial quality control pathologies and potential for hypoxic rescue.

In this Article, we show that hypoxia markedly extends lifespan in a mouse model of HtrA2 deficiency, establishing this mitochondrial disorder as responsive to hypoxia therapy. Mechanistically, HTRA2 loss leads to accumulation of aggregated IMS-facing C1 subunits, resulting in C1 destabilization and dysfunction. These findings align with our prior work in primary C1 disease and suggest that both primary and secondary C1 defects are amenable to hypoxia therapy. We propose that impaired IMS proteostasis in HTRA2 deficiency drives C1 instability, reducing mitochondrial oxygen consumption and causing pathological tissue hyperoxia. Extending this model, we identify the IMS disaggregase CLPB (Skd3, Suppressor of potassium (K) transport defect 3) as a key functional interactor of HTRA2. CLPB promotes aggregate resolubilization, whereas HTRA2 facilitates their degradation. Loss of CLPB similarly destabilizes C1 and may represent an additional cause of 3-MGA that is responsive to hypoxia. Together, these findings define a shared disease mechanism linking IMS proteostasis to C1 instability and provide a framework for identifying hypoxia-responsive mitochondrial disorders, from rare metabolic syndromes to more common neurodegenerative and age-associated diseases.

## Results

### Hypoxia rescues HTRA2 deficiency in vitro and in vivo

To identify mitochondrial disease genes amenable to hypoxia therapy, we previously performed a genome-wide CRISPR screen in K562 cells cultured under normoxia or hypoxia^8^. We focused on genes whose knockout (KO) caused a notable fitness defect in normoxia but not in hypoxia, suggesting they may be amenable to hypoxia therapy. By intersecting these hits with the Online Mendelian Inheritance in Man database, we nominated 75 candidate disease genes for hypoxia rescue. Many of these genes encode C1 subunits or assembly factors—consistent with our prior findings that C1 dysfunction is particularly responsive to hypoxia^6–8,25^. However, the screen also revealed diverse mitochondrial and non-mitochondrial hits, suggesting broader mechanisms of hypoxia rescue. We focused on HTRA2 owing to its disease relevance across multiple neurological syndromes and its strong effect size in the screen: all four *HTRA2*-targeting guide RNAs ranked among the top hypoxia-rescued hits, comparable to validated C1 subunits (Fig. 1b,c).

**Extended Data Fig. 1:**
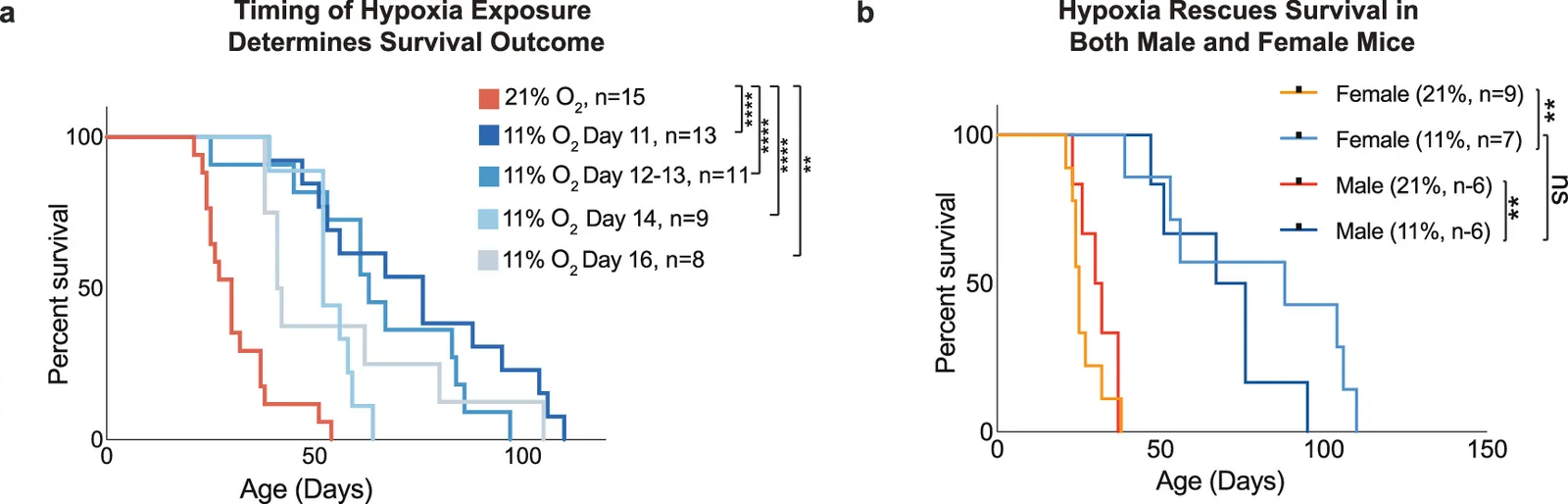
Effects of Timing and Gender for Hypoxia Therapy. **(a)** Kaplan–Meier survival curves of *Htra2* mutant mice exposed to hypoxia starting at different postnatal ages (P11–P16). Normoxia (21% O_2_), *n* = 15; Hypoxia (11% O_2_): starting at P11, *n* = 13; P12–13, *n* = 11; P14, *n* = 9; P16, *n* = 8. **(b)** Kaplan–Meier Survival Analysis of *Htra2* mutant mice exposed to hypoxia in males and females. p-values were calculated using the Log-rank (Mantel–Cox) test. *p < 0.05, **p < 0.005, ***p < 0.0005. Source data

To test whether HTRA2 deficiency is also amenable to hypoxia therapy in vivo, we leveraged a mouse model carrying a naturally occurring point mutation (S276C) in *Htra2*, commonly known as the mnd2 mutation, that abolishes the protease activity of HtrA2 (ref. ^21^). Homozygous mutants (hereafter referred to as *Htra2* mutant or *Htra2*^*M*^) display severe neurodegenerative phenotypes, including tremors, muscle atrophy, gait instability and early mortality within weeks after birth^20^. We exposed these mice to 11% oxygen starting at various postnatal days (P11–P16). Since exposure beginning at P11 led to the most pronounced increase in survival (Extended Data Fig. 1a), it was selected as the starting point for all subsequent experiments. Hypoxia led to a nearly threefold extension in median lifespan in both males and females and across two independent cohorts (Fig. 1d, Extended Data Fig. 1b and Supplementary Fig. 1), representing one of the most dramatic in vivo rescues of mitochondrial dysfunction we have observed—second only to our previous findings in *Ndufs4* KO mice. In addition to survival, hypoxia mildly rescued body weight (Fig. 1e) and significantly improved motor function as assessed by the inverted grid test (Fig. 1f) and rotarod test (Fig. 1g). To localize the brain regions most affected by HtrA2 loss and responsive to hypoxia, we stained for the inflammatory markers, Gfap and Iba1. We found that striatal inflammation was rescued by hypoxia, while inflammation in the thalamus was not (Fig. 1h and Supplementary Figs. 3 and 4). These results point to region-specific vulnerability and rescue, motivating future studies into the biochemical basis of this differential response.

### HTRA2 defects cause C1 instability in vitro and in vivo

**Fig. 2:**
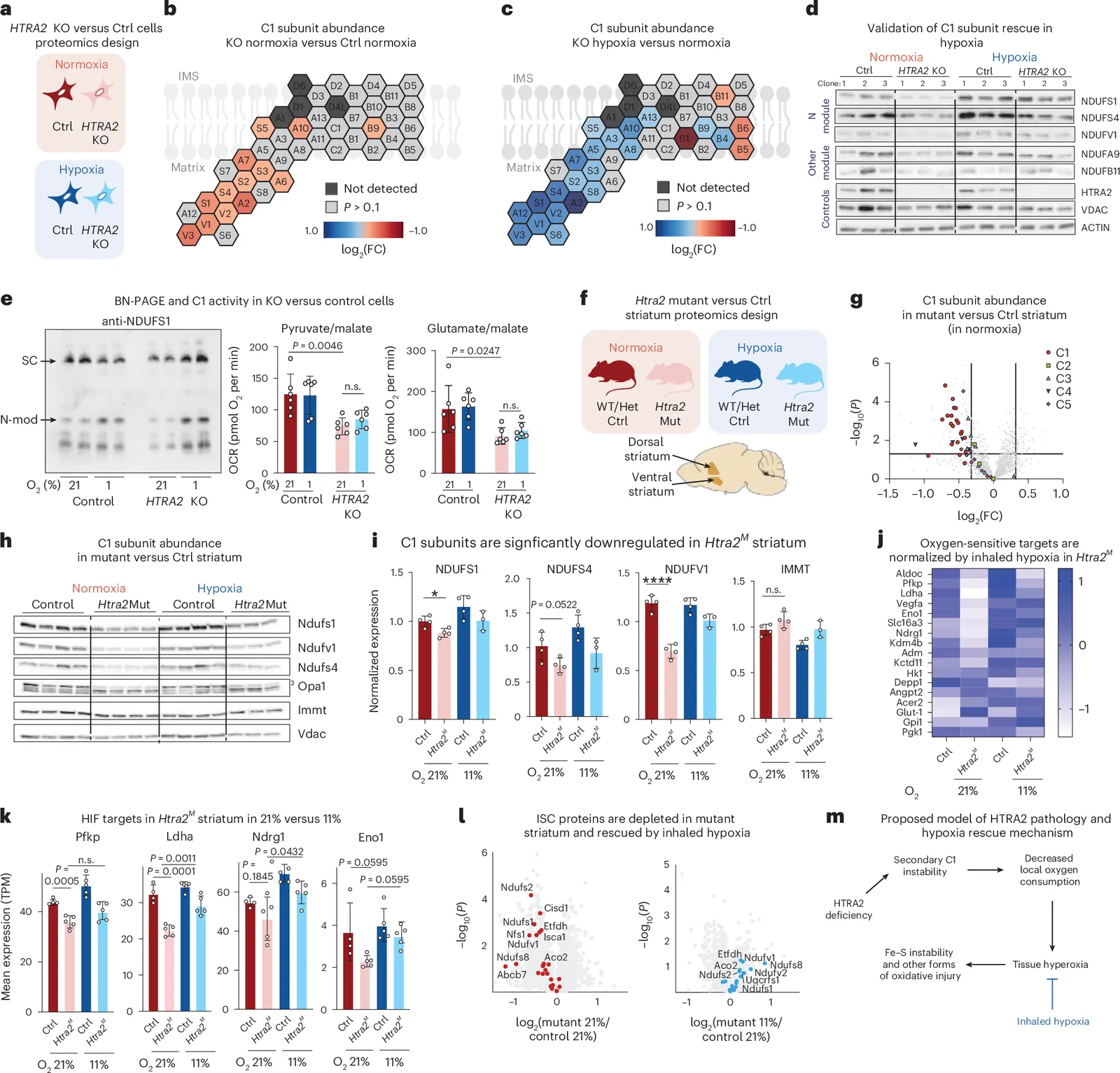
HTRA2 dysfunction leads to C1 instability. **a**, A schematic of the proteomics experiment in *HTRA2* KO and Ctrl cells. **b**,**c**, C1 subunit abundance in KO versus Ctrl cells. Hexagons represent individual subunits with colour reflecting log_2_fold change (FC) in KO versus Ctrl cells in normoxia (**b**) or in KO cells under hypoxia versus normoxia (**c**). **d**, Western blot validation of C1 subunit abundance across different modules in three single-cell clones of *HTRA2* KO or control cells. Samples are derived from the same experiment and gels/blots were processed in parallel. Data are representative of three biological replicates**. e**, BN-PAGE (left) and quantification of C1 activity (right) using different C1 substrates in KO versus Ctrl cells under normoxia or hypoxia for 3 days. Two to three clones per genotype were used. Data are presented as mean ± s.d. from six biological replicates. **f**, A schematic of striatum proteomics in *Htra2* mutant versus Ctrl mice in normoxia and hypoxia. **g**, A volcano plot of proteomics data from striatum of mutant versus Ctrl mice. C1–5 subunits highlighted with respective colours. (21% O_2_ control *n* = 5, mutant *n* = 5). An unpaired two-tailed *t*-test assuming equal variance was used to calculate unadjusted *P* values (*P* < 0.05). **h**, Western blot validation of proteomics findings in striatum from three to four mutant or control mice exposed to normoxia or hypoxia. Upper 2 bands in Opa1 signal are long and short forms of Opa1. **i**, Quantification of striatum western blot from four different cohorts after normalization with Vdac. Samples are derived from the same experiment and gels/blots were processed in parallel. Data are presented as mean ± s.d. from three to four biological replicates. **j**, A heat map from RNA-seq data (mean z-scores) showing expression of Hif target genes in Ctrl and *Htra2*^*M*^ mice maintained under normoxic (21% O_2_ control *n* = 4, mutant *n* = 5) or hypoxic (11% O_2_ control *n* = 5, mutant *n* = 5) conditions. **k**, Mean expression plots of selected canonical Hif target genes from the RNA-seq dataset. Data are presented as mean ± s.d. **l**, Volcano plots of striatum proteomics data showing depletion (left) of ISC proteins (red dots) in mutant striatum in normoxia and rescue (right) of ISC proteins (blue dots) by hypoxia. Axes range chosen to capture most relevant data (outliers not shown). An unpaired two-tailed *t*-test assuming equal variance was used to calculate unadjusted *P* values (*P* < 0.05). **m**, The proposed model of how hypoxia rescues C1 defects and tissue hyperoxia damage caused by HTRA2 deficiency. For bar plot data, *P* values were calculated by an unpaired two-tailed *t*-test. Het, heterozygous. Source data

**Extended Data Fig. 2:**
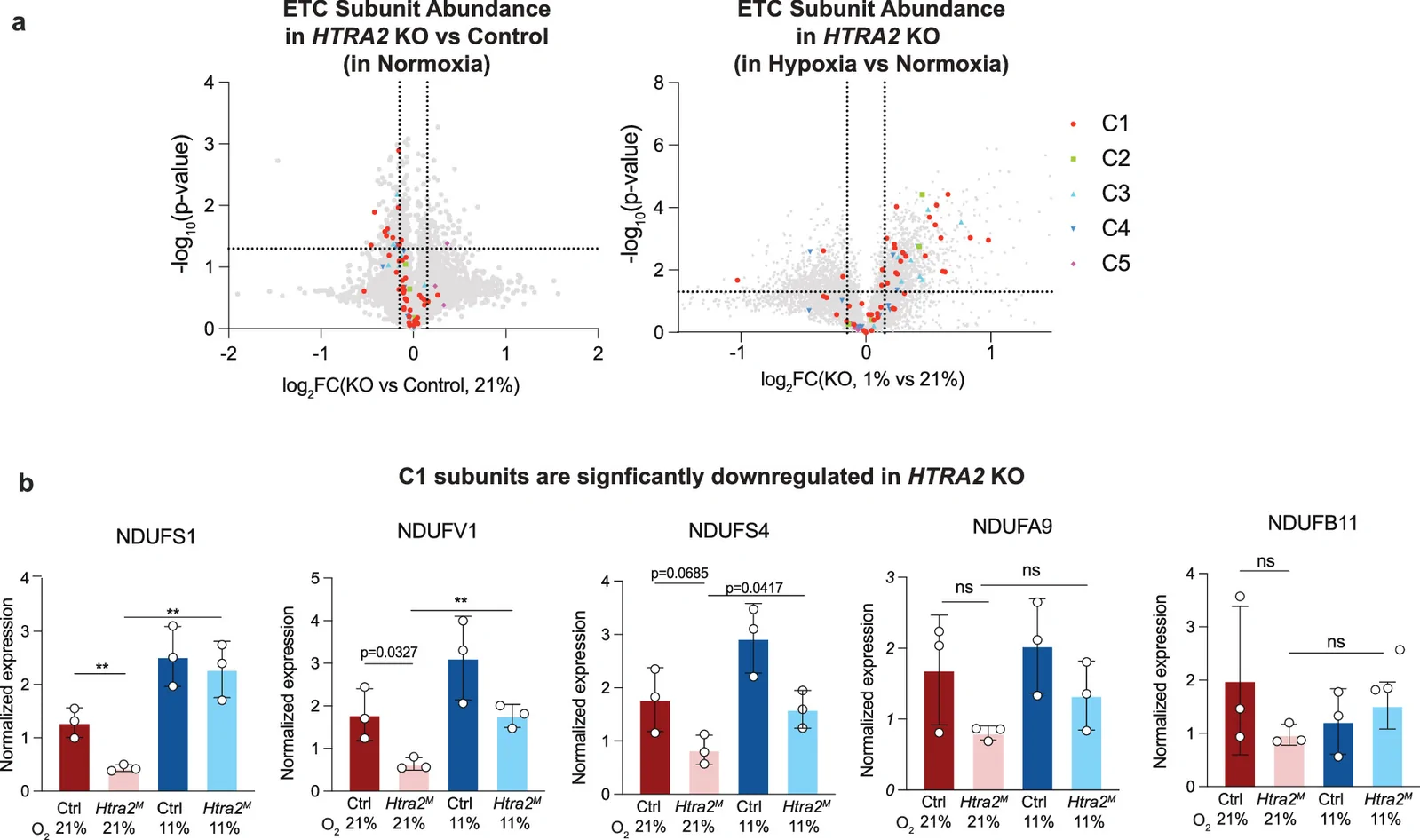
Differential regulation of respiratory chain subunits in *HTRA2* KO cells. **(a)** Volcano plots of quantitative proteomics from K562 cells highlighting Complex I–V (C1–C5) subunits. In normoxia (left), multiple C1–C5 subunits are downregulated in *HTRA2* KO cells relative to control and are upregulated in hypoxia relative to normoxia (right). Axes range chosen to capture most relevant data (outliers not shown). An unpaired two-tailed t-test assuming equal variance was used to calculate unadjusted p-values (p < 0.05). Dotted lines indicate raw p-value = 0.05 and raw fold-change (Mutant/Control) thresholds of 0.9 and 1.1. **(b)** Quantification of validation Western blots of C1 subunit abundance across different modules in three single-cell clones of *HTRA2* KO or control cells in Fig. 2d. Data are representative of three biological replicates and are presented as mean ± SD. Statistical significance was determined using unpaired two-tailed t-test. *p < 0.05, **p < 0.005. Source data

We next sought to define the molecular consequences of HTRA2 dysfunction. Given that HTRA2 is a protease, we performed proteomic analysis of *HTRA2* KO and control K562 cells cultured under normoxia or hypoxia for 3 days (Fig. 2a). *HTRA2* KO cells showed reduced abundance of ETC C1 subunits, particularly within the NADH-oxidizing N-module (Fig. 2b, Extended Data Fig. 2a and Supplementary Fig. 5). This is consistent with prior studies showing that the matrix-facing N-module is the most labile region of C1, with high turnover and sensitivity to proteostatic stress^26,27^. Hypoxia largely restored C1 subunit levels in *HTRA2* KO cells (Fig. 2c). These results were confirmed by western blot (Fig. 2d and Extended Data Fig. 2b).

To determine whether these protein-level changes resulted in improved ETC function, we performed blue native PAGE (BN-PAGE) and C1 activity assays using multiple substrates (Fig. 2e). These analyses confirmed that C1 assembly and activity are diminished in *HTRA2* KO cells and that hypoxia induced only modest increase in fully assembled C1, without improving enzymatic activity. These observations suggest that while hypoxia rescues C1 subunit stability, it may not restore its forward electron transfer capacity in this disease model. Given reduced C1 function, we next assessed whether *HTRA2* KO cells displayed reductive stress. We found no differences in NAD^+^/NADH between KO and control cells (Supplementary Fig. 2a). Consistent with this, expression of the yeast NADH dehydrogenase Ndi1—which bypasses C1 by transferring electrons from NADH to ubiquinone without proton pumping—did not rescue the fitness defect in *HTRA2* KO cells (Supplementary Fig. 2b). Together, these results suggest that reductive stress is unlikely to be the primary driver of the mutant phenotype or the hypoxia-mediated rescue.

We then investigated whether these C1 defects were also present in vivo. We focused specifically on the striatum, the brain region exhibiting significant neuroinflammation and pathology in *Htra2* mutant mice (Fig. 2f). We microdissected the striata of normoxic *Htra2* mutants and control littermates for proteomics and found that C1 subunit levels were markedly reduced in the mutants. Subunits of complexes III–IV were also moderately decreased, consistent with previous findings that C1 destabilization can trigger secondary losses in downstream ETC complexes (Fig. 2g and Extended Data Fig. 2a). Hypoxia exposure partially rescued the abundance of C1 subunit levels in vivo, mirroring the effect observed in cultured cells. These findings were validated by western blot (Fig. 2h,i).

While our results to this point show that C1 subunits are depleted in HTRA2 deficiency and the abundance is rescued by hypoxia, C1 activity was not obviously rescued. This was in line with our previous hypoxia CRISPR screen results where hypoxia rescued the majority of C1 KOs, including those that were directly involved in electron transfer reactions (for example, NDUFS1 and NDUFV1), already hinting at mechanisms that are beyond rescue of C1 activity.

We have previously shown that C1 loss reduces mitochondrial oxygen consumption, resulting in local tissue hyperoxia^7^. Elevated oxygen levels, in turn, destabilize key redox-sensitive molecules, including proteins containing ISCs^2,28,29^. We therefore wondered whether loss of HtrA2 also causes tissue hyperoxia. Direct measurement of tissue partial oxygen pressure (pO_2_) in the mouse striatum proved technically challenging. As a proxy for tissue hyperoxia, we found that ISC-containing proteins were depleted in the *Htra2* mutant striatum under normoxia and this was reversed by hypoxia (Fig. 2l), supporting the hypothesis that tissue hyperoxia contributes to disease and that hypoxia mitigates this stress.

As an orthogonal approach, we performed RNA-sequencing (RNA-seq) analysis of striatal tissue from *Htra2* mutant and control mice exposed to normoxia or hypoxia. Our analysis revealed that canonical HIF target genes were downregulated in *Htra2* mutant striatum under normoxia, consistent with a relatively hyperoxic state. Importantly, these targets were largely normalized following hypoxia exposure (Fig. 2j,k). Admittedly, these results are simply suggestive of tissue hyperoxia, since both ISC proteins and HIF targets are only indirect measures for local oxygen tensions. Nonetheless, these data suggest that HTRA2 deficiency causes a secondary C1 defect that impairs mitochondrial oxygen consumption, probably leading to localized tissue hyperoxia and downstream oxygen toxicity (Fig. 2m). Inhaled hypoxia appears to reverse these defects by restoring oxygen homeostasis, particularly within vulnerable brain regions. While ISC restoration serves as a compelling readout of rescue, the incomplete recovery of C1 activity suggests that additional downstream consequences of hyperoxia remain to be defined.

### HTRA2 is functionally related to mitochondrial IMS protein disaggregase, CLPB

We next reasoned that other proteins involved in mitochondrial proteostasis, particularly those that interact functionally with HTRA2, may show similar response patterns to hypoxia. To identify candidate proteins, we turned to the Dependency Map (DepMap) dataset, a large-scale resource of CRISPR-based gene essentiality profiles across hundreds of cell lines. DepMap identifies genes with similar functional roles based on shared patterns of dependency across diverse cellular contexts^30^.

**Fig. 3:**
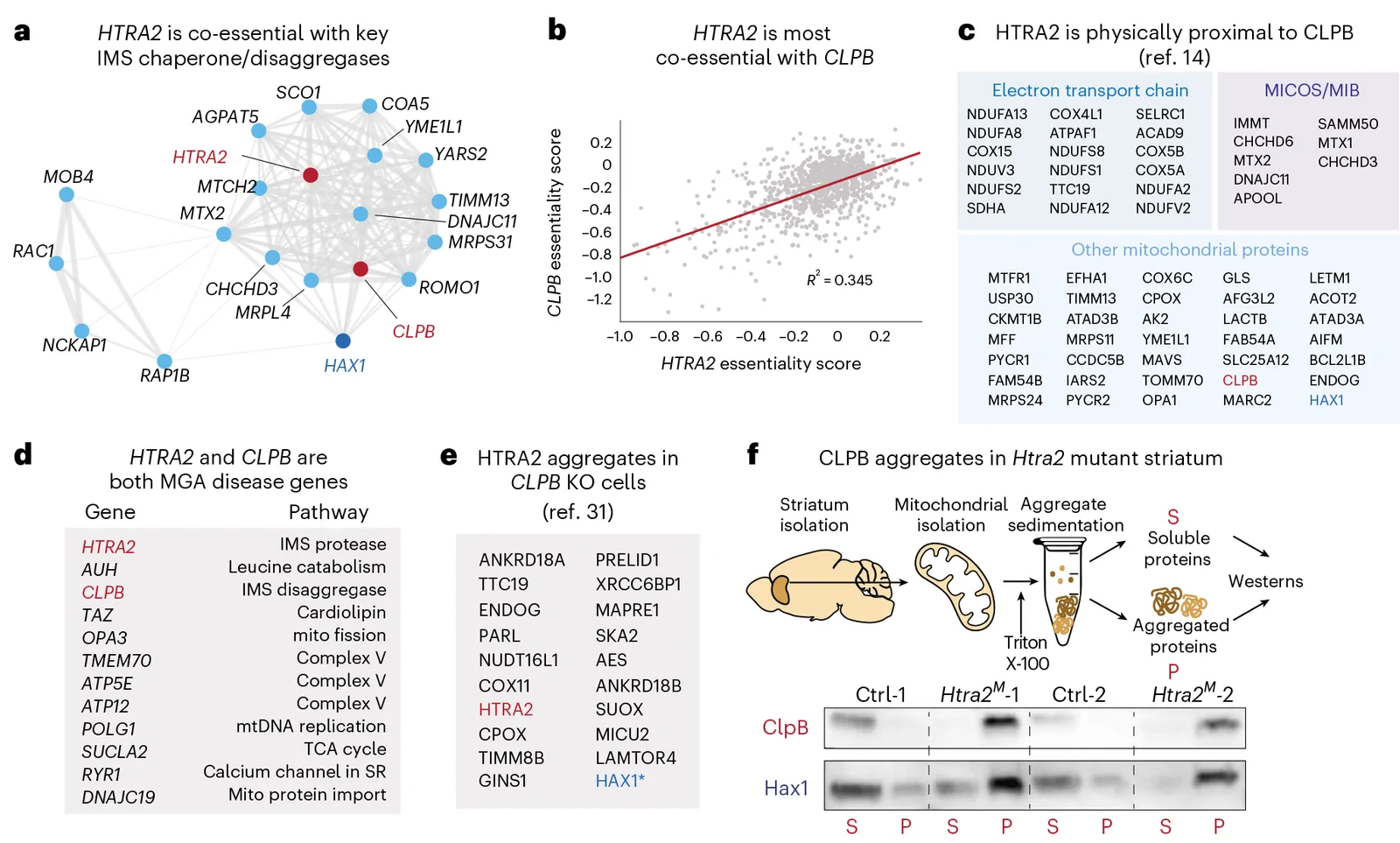
HTRA2 and CLPB are functionally related. **a**, The *HTRA2* co-dependency network as defined by FIREWORKS analysis of DepMap datasets. **b**, DepMap essentiality scores for *CLPB* versus *HTRA2* reveal substantial co-dependency across numerous cancer cell lines. Axes range chosen to capture most relevant data (outliers not shown). *R*^2^ was calculated by simple linear regression model^30^. **c**, Proteins found to be physically proximal to HTRA2 based on a previously published BioID dataset^14^. **d**, A list of known 3-MGA causing disease genes. **e**, A list of top proteins that aggregate in *CLPB* KO cells based on previously published work^31^. Asterisk indicates rank 72. **f**, ClpB and Hax1 aggregate in striatal mitochondria from *Htra2* mutant mice. Top: mitochondria were isolated from microdissected striata and soluble versus aggregated fractions separated for analysis. Bottom: equal amounts of protein across samples were subjected to fractionation and western blot analysis (*n* = 2 mice per group). Data are representative of two biological replicates. S, soluble; P, pellet (aggregate). Source data

Among the top genes co-essential with *HTRA2* were several mitochondrial quality control genes, including *CLPB*, *HAX1*, *YME1L1*, *DNAJC11* and *MTX2*—all with known or predicted roles in protein folding, degradation or mitochondrial stress responses (Fig. 3a). We further refined our analysis to identify bidirectional co-essential pairs, where each appears as a top co-essential hit in the analysis of the other gene. Notably, *HTRA2* and *CLPB* emerged as a strong bidirectional co-essential pair, with highly ranked and symmetric scores suggestive of a robust functional relationship (Fig. 3b). CLPB, also known as Skd3, is a metazoan mitochondrial protein disaggregase localized to the IMS^31^. Another top co-essential gene with both *HTRA2* and *CLPB* was *HAX1*. HAX1 has been reported to be required for processing HTRA2 to its active form^32^.

To assess whether these co-essentiality signals correspond to physical interactions, we re-examined previously published BioID proximity labelling datasets^14^. Both CLPB and HAX1 were found in HTRA2 BioID interactomes, suggesting their possible physical association (Fig. 3c). Importantly, *CLPB* is also a known 3-MGA disease gene^33^, further linking it to the phenotypic spectrum shared with *HTRA2* (Fig. 3d). Taken together, these orthogonal lines of evidence—co-essentiality, physical proximity and overlapping disease relevance—strongly support a model in which HTRA2 and CLPB act together in maintaining mitochondrial IMS proteostasis.

We next tested whether these proteins might be required for one another’s solubility, given their physical interaction. We microdissected striata from *Htra2* mutant and control mice, isolated mitochondria, lysed, separated soluble and aggregated fractions by ultracentrifugation and analysed by western blotting (Fig. 3f). Both ClpB and Hax1 showed increased aggregation in *Htra2*^*M*^ relative to control striatum. Previous studies have similarly shown that HTRA2 and HAX1 aggregate in *CLPB* KO cells, suggesting mutual dependence for proper folding and stability^31,34^ (Fig. 3e). These results support a model in which HTRA2 and CLPB function as a coordinated network in IMS proteostasis, with potential direct physical interactions and interdependence for stability.

### CLPB competes with HTRA2 for aggregated substrates and is also required for C1 stability

**Fig. 4:**
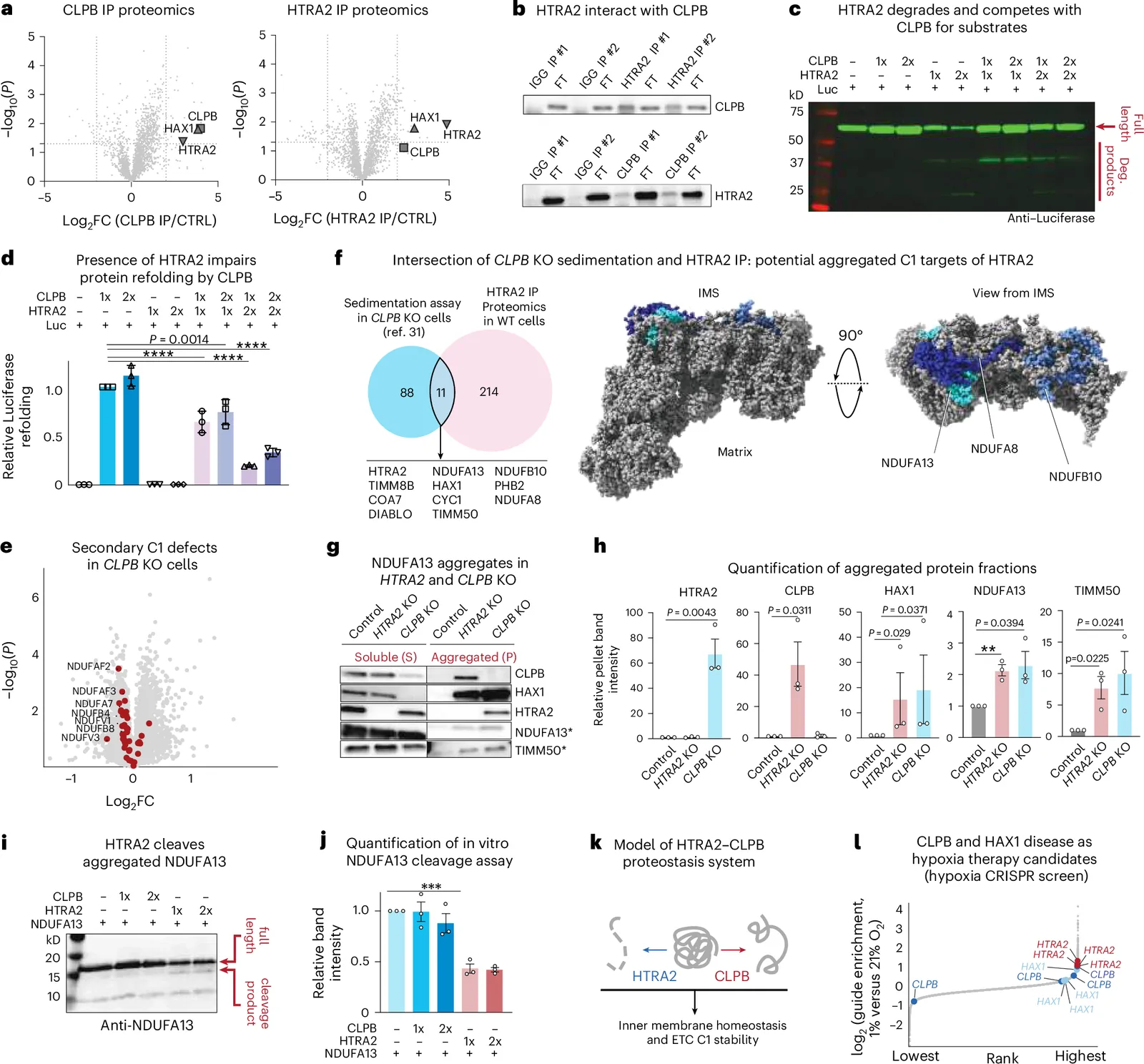
HTRA2 and CLPB coordinate IMS homeostasis and C1 stability. **a**, A volcano plot for IP proteomics against HTRA2 (right) or CLPB (left) compared to IgG in HEK cells. CLPB, HTRA2 and HAX1 are all hits in both IP experiments. Axes range chosen to capture most relevant data (outliers not shown). An unpaired two-tailed *t*-test assuming equal variance was used to calculate unadjusted *P* values (*P* < 0.05). **b**, Physical interaction was validated by IP followed by western blotting in HEK cells. Pull-downs were performed using IgG, HTRA2 and CLPB antibodies. HTRA2 was detected in the CLPB immunoprecipitated fraction and vice versa. Data are representative of three biological replicates. FT, flow through. **c**, Aggregated luciferase was incubated with purified CLPB and/or HTRA2 for 60 min, followed by SDS–PAGE western blot using luciferase antibody to detect full length as well as degraded (deg.) luciferase products. Data are representative of three biological replicates. **d**, Aggregated luciferase was incubated with HTRA2 and/or CLPB for 60 min, followed by measuring luciferase activity. Data are plotted as mean ± s.e.m. from three biological replicates. A one way ANOVA was used to calculate *P* values. **e**, A volcano plot of proteomics data from *CLPB* KO versus control cells (*n* = 4 per group). C1 subunits are shown in red. Axes range chosen to capture most relevant data (outliers not shown). An unpaired two-tailed *t*-test assuming equal variance was used to calculate unadjusted *P* values (*P* < 0.05). **f**, Left: reanalysis of a published sedimentation assay^31^ in *CLPB* KO cells intersected with our HTRA2 IP proteomics data to identify common targets of the HTRA2/CLPB proteostasis system. Right: ChimeraX visualization of C1 proteins overlapping between CLPB aggregation proteomics and HTRA2 IP datasets shows that NDUFA13, NDUFB10 and NDUFA8 are at least partially exposed to the IMS. **g**, Western blot analysis of soluble versus insoluble mitochondrial fractions from *CLPB* KO and *HTRA2* KO cells (asterisks indicate the exposure times were optimized for soluble and pellet fractions separately). Data shown are representative of three biological replicates. **h**, Quantification of the pellet band intensities normalized to control, across three biological replicates. Data are presented as mean ± s.e.m. *P* values were calculated using an unpaired two-tailed *t*-test. **i**, NDUFA13 aggregates were generated and purified CLPB or HTRA2 were added as indicated. Reactions were analysed by western blot using an anti-NDUFA13 antibody. Data shown are representative of three biological replicates. **j**, Quantification of the cleavage assay from three biological replicates, measuring full-length band intensities across different conditions normalized to full-length NDUFA13. Data are presented as mean ± s.e.m. *P* values were calculated using an unpaired two-tailed *t*-test. **k**, The proposed model of HTRA2 and CLPB’s roles in the IMS. **l**, *HTRA2*, *CLPB* (and *HAX1*) gRNAs also show hypoxia rescue in previous K562 CRISPR screens, suggesting possible disease candidates for hypoxia therapy. ****P < 0.0001. Source data

To more directly probe the functional relationship between HTRA2 and CLPB, we first performed immunoprecipitation (IP) followed by mass spectrometry-based proteomics under endogenous conditions. In line with the BioID results, HTRA2 and CLPB were among the top interactors in each other’s IP datasets, supporting a strong physical association (Fig. 4a). These interactions were independently validated by co-IP and western blotting (Fig. 4b).

We next asked whether CLPB modulates the proteolytic activity of HTRA2. To this end, we incubated recombinant HTRA2 with aggregated luciferase in the presence or absence of recombinant CLPB. As expected, HTRA2 alone was capable of degrading luciferase, consistent with its protease activity (Fig. 4c). Interestingly, the addition of CLPB appeared to partially protect luciferase from degradation by competitively binding aggregates. We then tested the reverse question—whether HTRA2 impacts CLPB’s disaggregase function. Using a standard luciferase reactivation assay, we found that CLPB alone was sufficient to disaggregate aggregated luciferase and restore enzymatic activity (Fig. 4d). However, co-incubation with HTRA2 dampened this recovery in a dose-dependent manner, probably due to substrate degradation by HTRA2. These findings suggest that HTRA2 and CLPB can act on an overlapping set of aggregated substrates—one acting to disaggregate, the other to degrade—to maintain IMS proteostasis.

To understand whether this interplay impacts mitochondrial function more broadly, we performed proteomics of *CLPB* KO cells. We observed widespread depletion of C1 subunits (Fig. 4e), mirroring the defects seen in *HTRA2* KO cells. This convergence on secondary C1 instability underscores a shared role of HTRA2 and CLPB in mitochondrial protein quality control. Prior studies have shown that C1 subunits can form insoluble aggregates in *CLPB* KO cells^35^, raising the possibility that HTRA2 and CLPB are jointly required to manage aggregated or misfolded forms of C1 subunits at the IMS–inner membrane interface or during their transit across the IMS (Fig. 4k).

### HTRA2–CLPB proteostasis controls NDUFA13 solubility and C1 stability

To further explore the mechanistic link between HTRA2/CLPB and C1 subunits, we intersected (1) proteins that aggregate in the *CLPB* KO condition^31^ with (2) proteins identified in HTRA2 IP proteomics. This comparative analysis revealed several common hits, including multiple C1 subunits (NDUFA13, NDUFA8 and NDUFB10) and mitochondrial protein import machinery subunits (for example, TIMM50) (Fig. 4f). Interestingly, all three C1 subunits identified reside in the IMS-facing portion of assembled C1, where HTRA2 and CLPB are localized. Of note, previous proximity proteomics datasets of the IMS have detected all these proteins (NDUFA13, NDUFA8, TIMM50), indicating that they are exposed to the IMS^36^. We focused on NDUFA13 as a representative C1 subunit that is directly affected by loss of the HTRA2–CLPB proteostasis system.

To test whether C1 subunits aggregate upon HTRA2 and CLPB loss, we performed sedimentation assays to separate soluble and aggregated proteins. Specifically, we generated *HTRA2* and *CLPB* KO cells and separated soluble versus aggregate (pellet) fractions, followed by western blots. We first confirmed that HTRA2 and HAX1 aggregated in *CLPB* KO cells and, conversely, CLPB and HAX1 aggregated in *HTRA2* KO cells (Fig. 4g,h), in line with a potential HTRA2–CLPB–HAX1 protein complex that is stabilized by the presence of all three components. Importantly, NDUFA13 aggregated in both *HTRA2* and *CLPB* KO cells, thereby providing a direct link between IMS and C1 proteostasis. We additionally observed aggregation of TIMM50, which may also affect import of C1 subunits (Fig. 4g,h). To determine whether HTRA2 directly recognizes C1 subunit aggregates, we performed in vitro cleavage assays using purified, aggregated NDUFA13. We found that HTRA2 can directly recognize and cleave aggregated NDUFA13 in line with previous results^37^ (Fig. 4i,j).

Together, these findings support a model in which HTRA2 and CLPB cooperate to maintain C1 stability by preserving key subunits such as NDUFA13 in a soluble, assembly-competent state. Loss of this proteostatic pathway leads to NDUFA13 aggregation, impaired C1 assembly and secondary loss of C1 subunits. Our data support a model in which HTRA2 and CLPB directly act on IMS-facing C1 subunits. However, it is also conceivable that loss of HTRA2 and CLPB indirectly disrupts C1 via broader architectural changes in the mitochondria—such as altered cristae structure or impaired membrane dynamics—which are known to affect respiratory chain assembly^14,38,39^. Consistent with this hypothesis, we also found that HTRA2 was co-essential and physically proximal to numerous components of the MICOS complexes, which have been found to bridge the outer and inner membranes, enabling cristae formation and stabilizing the ETC within cristae (Supplementary Fig. 6). Likewise, loss of CLPB has been shown to result in altered mitochondrial cristae morphology and a decrease in mitochondrial fusion dynamics^40^. Similarly, we observed impaired cristae morphology in the mitochondria of *Htra2* mutant brain tissue (Supplementary Fig. 7). Regardless of the precise mechanism, our data demonstrate that both *HTRA2* and *CLPB*, two 3-MGA disease genes, converge on a shared biochemical phenotype of C1 destabilization.

Given our central hypothesis that hypoxia can rescue secondary C1 defects, we next revisited our original CRISPR screen to assess whether *CLPB* KO cells exhibit hypoxia responsiveness. Indeed, *CLPB* and *HAX1* were also among the top hits whose fitness defects in normoxia were rescued under hypoxia (Fig. 4l). This finding suggests that, like HTRA2, CLPB (and HAX1) deficiency may also be amenable to hypoxia therapy. These results open the door to extending hypoxia-based treatments to additional forms of 3-MGA and other disorders of mitochondrial protein quality control.

## Discussion

We previously demonstrated that hypoxia dramatically extends lifespan and reverses neurological deficits in the *Ndufs4* KO mouse, a model of primary C1 disease^6^. Our initial CRISPR screen identified numerous C1-related genes whose loss-of-function could be rescued by hypoxia but also revealed a broader set of hits beyond canonical ETC subunits^8^. In the current study, we identified key regulators of mitochondrial proteostasis, such as *HTRA2* and *CLPB*, as additional genes whose loss is amenable to hypoxia therapy. We propose that the shared underlying mechanism in these diverse hypoxia-rescuable conditions is C1 dysfunction. This dysfunction leads to impaired oxygen consumption, resulting in a state of local tissue hyperoxia. This hyperoxic environment is a pathogenic state that can be effectively reversed by lowering the ambient oxygen tension. This framework opens the door for reanalysing prior CRISPR screens with a focus on identifying other regulators of C1 function or stability and motivates future targeted screens to uncover additional hypoxia-rescuable conditions.

However, other forms of ETC dysfunction may not be equally responsive to hypoxia. While C1 defects allow for partial compensation via anaerobic glycolysis and alternative redox pathways for NADH oxidation, severe dysfunction of complexes II–IV could have broader metabolic consequences, such as disrupting TCA cycle flux or impairing proton motive force generation in ways that are not bypassed by hypoxia^41–44^. Similarly, while hypoxia provides impressive symptomatic relief in the *Fxn* KO mouse, it does not extend lifespan, probably because *FXN* deficiency disrupts multiple essential processes beyond C1 stability, including ISC biogenesis^29^. Thus, hypoxia therapy may be most effective for diseases centred on C1 dysfunction, where the major pathological driver is local hyperoxia and its downstream consequences. Nevertheless, our findings extend the concept of hypoxia therapy from the limited set of disease-associated mutations in C1 subunit-encoding genes to the much broader class of indirect regulators of C1 stability.

Mitochondrial protein quality control is disrupted in a wide range of diseases, including rare inborn errors such as 3-methylglutaconic aciduria (for example, due to mutations in *HTRA2*, *CLPB*, *DNAJC19* or *TIMM50*), as well as common neurodegenerative disorders such as Parkinson’s disease, Alzheimer’s disease and amyotrophic lateral sclerosis^4^. In each of these conditions, the accumulation of misfolded or aggregated proteins within mitochondria may contribute to pathology. The observation that hypoxia can partially or fully rescue disease in HTRA2 deficiency indicates that mitochondrial proteostasis defects may in fact be modifiable by oxygen tension.

To assess the effects of chronic hypoxia, we previously characterized whole-body physiological changes. Many acute hypoxia effects (for example, behaviour, temperature and food intake) normalize with chronic exposure, yet the hypoxia rescue effect persists across both timescales^45^. Notably, HIF activation alone does not rescue the *Ndufs4* KO mouse model, indicating that canonical hypoxia responses are insufficient to explain the benefit^25^. While hyperoxia probably contributes to pathology, other physiological factors cannot be excluded. While our data highlight C1 destabilization as a key driver of HTRA2-related disease and target of hypoxia rescue, additional aspects of proteotoxic stress and oxidative damage may also be directly mitigated by hypoxia. Furthermore, hypoxia may influence mitochondrial proteostasis through modulation of translation rates, reducing the influx of nascent proteins into already stressed organelles, or by altering mitochondrial protein import dynamics^46^. We have also previously shown that hypoxia affects protein turnover rates, including for ISC-containing proteins, which are particularly vulnerable to oxidative degradation^2^. These multifaceted effects suggest that hypoxia may provide a global reprieve from proteostatic stress by tuning mitochondrial protein import, processing and degradation pathways. Future work will be required to dissect the relative contribution of these mechanisms to disease rescue.

Importantly, the impact of impaired mitochondrial proteostasis in HtrA2 deficiency is likely to be specific to cell type and brain region. For example, we observed an inflammatory phenotype in the striatum and thalamus of mutant mice, but not in other regions, such as the cortex or hippocampus. Moreover, we found hypoxia rescued striatal lesions, but not those in the thalamus. These regional differences may be influenced by a range of variables such as differences in aggregate burden, compensatory proteostasis systems, ETC C1 demand or local oxygen delivery.

Our findings, together with emerging studies, support a close functional and physical relationship between HTRA2 and CLPB in maintaining mitochondrial IMS proteostasis^14,31,34^. CLPB functions as a disaggregase and HTRA2 as a protease, and our biochemical assays suggest they compete for overlapping substrates. Moreover, they probably act on IMS-facing C1 subunits, such as NDUFA13. Given that NDUFA13 loss is sufficient to destabilize C1 (ref. ^47^), these findings provide a mechanistic link between impaired HTRA2/CLPB activity and C1 dysfunction. We think that HTRA2 does not simply regulate NDUFA13 abundance through bulk degradation but instead functions in a quality control pathway that removes non-productive NDUFA13 species. During import or C1 assembly, a fraction of NDUFA13 may misfold or fail to incorporate into productive assembly intermediates. In wild-type (WT) cells, HTRA2-mediated proteolysis would eliminate these aberrant molecules before they accumulate. In the absence of HTRA2, however, these species persist and form aggregates, which may not only divert NDUFA13 away from the assembly pathway but also exert toxic effects by sequestering newly imported NDUFA13 or interacting C1 subunits, thereby interfering with C1 assembly. Thus, by removing misfolded NDUFA13 before it can disrupt assembly, HTRA2 may act as a proteostatic safeguard that preserves efficient C1 biogenesis and activity. In addition, reduced TIMM50 solubility may impair mitochondrial protein import and proper assembly of C1 subunits, as C1 assembly relies on the TIM23, TIM22 and MIA pathways^48^.

While this work focused primarily on HTRA2 and CLPB, HAX1 also emerged as a key player in this network. Mutations in *HAX1* cause a clinically similar mitochondrial disorder and, notably, *HAX1* was a hit in our hypoxia CRISPR screen. Functionally, HAX1 is required for the proteolytic processing and activation of HTRA2 (ref. ^32^), and its deficiency probably impairs HTRA2 function. Given its highly disordered structure, HAX1 may also serve as a substrate adaptor, helping to deliver aggregated proteins to HTRA2 and CLPB for clearance or disaggregation. Together, these data support a model in which HTRA2, CLPB and HAX1 form a cooperative quality control module in the IMS. Disruption of any of these components can lead to proteostasis collapse, C1 instability, and disease—a pathway that may be broadly amenable to rescue by hypoxia.

Beyond elucidating the molecular interplay between HTRA2, CLPB and HAX1, our long-term goal is to translate these insights into therapeutic strategies. To this end, we recently developed a small molecule that mimics the effects of hypoxia, HypoxyStat^49–52^. This compound functions by increasing the binding affinity of oxygen and haemoglobin, thereby preventing the release of oxygen into tissues and resulting in lower brain oxygen levels. Importantly, HypoxyStat has shown efficacy in vivo (in the primary C1 disease model—*Ndufs4* KO mice) when administered starting around postnatal day 30 (P30), a window that is later than what is required for effective rescue of *Htra2* mutant mice. Next-generation HypoxyStat derivatives will allow for earlier intervention. This will enable future clinical trials targeting mitochondrial proteostasis disorders using systemically deliverable small molecules that decrease oxygen delivery.

## Methods

### Animal use and care

*Htra2* mutant mice (mnd2; strain no. 004608, background: C57BL/6J) were obtained from The Jackson Laboratory. Breeding was performed to generate homozygous mutants and corresponding controls. Both WT and heterozygous littermates were included in experiments, as heterozygotes are phenotypically normal. Pups were weaned and genotyped approximately 21 days postnatally. No sex-based bias was introduced in the study design. All animals were housed in a temperature-controlled facility maintained at 20–22 °C with 30–70% relative humidity under a 12-h light/dark cycle (lights on at 07:00). All animals had ad libitum access to standard chow diet (5053, PicoLab Rodent Diet 20) and water, with additional feed and hydrated gel packs added to cages of newly weaned pups. All animal procedures were approved by the Institutional Animal Care and Use Committee (IACUC) at UCSF/Gladstone Institutes.

### In vivo hypoxia exposure and survival curves

The hypoxia chamber setup is as previously described^49^. Mice were housed in 472 L acrylic boxes. Eleven per cent FiO_2_ was obtained by mixing a constant flow of nitrogen and air generated by splitting room air using a LABTEK 4CPx-P nitrogen generator. Soda lime was added to the chambers as a CO_2_ scavenger. The oxygen concentration was measured at the outlet port of the chamber with an O_2_ sensor (Vernier), which was calibrated monthly. Starting at postnatal day 11, pups were transferred to the appropriate oxygen condition along with their mother (they were not weaned until ~P21), and the number of pups was recorded three times per week. If any pup was found deceased, tissue from the carcass was collected for genotyping. Between P16 and P18, pups were ear tagged for identification and genotyped, with body weights recorded three times weekly. Monitoring continued until natural death, scheduled euthanasia, or euthanasia for tissue collection.

### Brain immunofluorescence

Mice (~4 weeks old) were transcardially perfused with PBS and 4% PFA (10 ml each). Brains were post-fixed overnight in 4% PFA at 4 °C, washed in PBS, cryoprotected in 30% sucrose/PBS and frozen at −20 °C. Coronal sections (40 µm) were collected serially in cryoprotectant. Free-floating sections were permeabilized in 0.2% Triton X-100/PBS and blocked in 4% FBS (1 h, room temperature). Sections were incubated with primary antibodies overnight, followed by secondary antibodies (2 h to overnight, room temperature) and mounted on positively charged slides. The primary antibodies used were IBA1 (1:250, Genetex GTX100042, RRID:AB_1240434) and GFAP (1:1,000, Sigma MAB3402, RRID: AB_94844). Secondary antibodies used were donkey anti-mouse-594 (1:250, Invitrogen A32744, RRID: AB_2762826) and donkey-anti-rabbit-488 (1:250, Invitrogen A21206, RRID:AB_2535792). Imaging was performed using a Leica slide scanner, and lesion quantification across samples was conducted by blinded scoring.

### Inverted grid test

A wire grid (1 cm × 1 cm, for example, cage lid) was securely positioned above soft bedding or foam padding to prevent injury from falls. Mice (~4 weeks old) were placed on the grid and gently inverted 180°, allowing the animal to hang upside down. The latency to fall was recorded. Three trials were performed per mouse with 5-min rest intervals. Trials in which the mouse jumped intentionally were excluded or repeated. The average hang time was calculated per mouse.

### Rotarod test

Motor coordination and balance were assessed using an accelerating rotarod apparatus. Mice (~4 weeks old) were placed on a rotating rod that accelerated from 4 to 40 rpm (5 rpm per min) and latency to fall was recorded automatically. Each mouse performed three trials per session with at least 10 min between trials to minimize fatigue. The average latency to fall was used for statistical analysis.

### Cell lines

K562 (CCL-243; RRID: CVCL 0004, ATCC authenticates cell line identity before distribution) and HEK293T (from UCSF Cell and Genome Engineering Core, validated by STR profiling, confirmed negative for mycoplasma contamination by PCR (ATCC, 30–1012K)) were used in this study. These were cultured in complete medium (DMEM; Gibco, 11995073) supplemented with 10% FBS (Corning, MT35015CV) and 1% penicillin–streptomycin–glutamine (Corning, MT30009CI). Cells were maintained by passaging before reaching a confluency of 1 × 10^6^ cells ml^−1^. To generate KO cells, sgRNAs were cloned into the lentiCRISPRv2 vector. Lentiviral particles were produced by co-transfecting HEK293T cells with the sgRNA-containing lentiCRISPRv2 plasmid together with the packaging plasmids psPAX2 and VSV-G using X-tremeGENE transfection reagent. Viral supernatants were collected and used to transduce target cells, which were subsequently selected with puromycin for 72 h to enrich for successfully transduced cells.

### K562 proteomics sample preparation, mass spectrometry and data analysis

K562 cells (*n* = 2, control and *n* = 3, *HTRA2* KO) were seeded at 1 × 10^5^ cells ml^−1^ in six-well plates and cultured under normoxic (21% O_2_) or hypoxic (1% O_2_) conditions. Cells were collected at day 3, washed with PBS and flash-frozen for TMT proteomics at the Sanford Burnham Proteomics Core. In brief, cells were lysed in 8 M urea/50 mM ammonium bicarbonate with Benzonase, proteins quantified by the BCA assay, then reduced, alkylated and digested with trypsin/Lys-C. Peptides were desalted, TMT10plex-labelled, pooled and fractionated by high-pH reversed-phase chromatography before nanoLC–MS/MS analysis on a nanoACQUITY–Orbitrap Fusion Lumos system (74-min gradient). Data were processed in MaxQuant (v1.5.5.1) against the human UniProt database (1% false discovery rate (FDR)), with carbamidomethylation as fixed and methionine oxidation/N-terminal acetylation as variable modifications. Reporter intensities were log_2_-transformed and locally estimated scatterplot smoothing normalized. Differential abundance analysis was performed using g:Profiler and R (v4.6.0).

### HEK proteomics sample preparation, mass spectrometry and data analysis

HEK293T cells (*n* = 4 across control, *CLPB*, *HTRA2* and *HAX1* KO conditions) were lysed in modified RIPA buffer (10% SDS, 50 mM ammonium bicarbonate and Halt Protease Inhibitor) with Benzonase (15–20 min, room temperature), sonicated (10 cycles), and centrifuged at 20,000*g* for 15 min at 4 °C. Protein concentrations were determined by the BCA assay and lysates were sent for proteomics to UC Davis Proteomics Core. In brief, proteins were processed by S-Trap digestion (DTT reduction, iodoacetamide alkylation, trypsin 1:100) and analysed by nanoLC–DIA-PASEF on an Evosep One–timsTOF HT system (*m*/*z* 350–1,191). Data were searched in Spectronaut (DirectDIA) against the human UniProt database (1% FDR, ≤2 missed cleavages), with carbamidomethylation as fixed and methionine oxidation/N-terminal acetylation as variable modifications and normalized by local regression.

### Striatum tissue proteomics

Striata were microdissected from frozen hemi-brains of ~4-week-old mice (n = 5 per group) sectioned coronally at 400–500 µm using a McIlwain Tissue Chopper after embedding in 0.6% low-melting-point agarose (Invitrogen, #15517-014). Sections were transferred into PBS with protease inhibitors (Roche, #05892953001), striata dissected under a microscope, and flash-frozen at –80 °C. Samples were prepared using the S-Trap Mini Kit (K002-MINIX-0010KT) and sent to UC Davis Proteomics Core. Briefly, proteins were quantified by BCA assay, processed by S-Trap digestion (DTT reduction, iodoacetamide alkylation, trypsin 1:100, 37 °C overnight), and eluted peptides analysed by nanoLC–PASEF on a nanoElute–timsTOF Pro system (30-min gradient). Data were searched in FragPipe (v19) against the mouse UniProt database (1% FDR, ≤2 missed cleavages), with carbamidomethylation as fixed and N-terminal acetylation, methionine oxidation and deamidation (N/Q) as variable modifications. Label-free quantification was performed using IonQuant with match-between-runs enabled.

### SDS−PAGE westerns

Brain tissues previously stored at –80 °C were retrieved on dry ice and lysed in 100–200 µl of freshly prepared RIPA buffer (volume adjusted based on tissue size to achieve a near-transparent lysate). Homogenization was performed using a motorized homogenizer for 1 min (30 s on, 30 s off), followed by sonication for 5 min (30 s on, 30 s off). Lysates were then centrifuged and the supernatant was collected. Protein concentration was measured using 1 µl of lysate with the BCA assay.

For cell lysates, frozen cell pellets were resuspended in 100 µl RIPA buffer and incubated on ice for 15–30 min with intermittent mixing. Lysates were centrifuged at maximum speed for 15 min at 4 °C and the supernatant was transferred to fresh tubes for the BCA assay. Samples at equal concentrations were prepared in 1× Laemmli buffer and boiled at 95 °C for 5 min. Proteins were separated on 8–16 % gradient Bio-Rad SDS–PAGE gels and transferred onto PVDF membranes using a semi-dry transfer apparatus. For blocking buffer, 2% milk in TBST was used. The primary antibodies used were NDUFS1 (1:10,000, Abcam ab169540, RRID: AB_2687932), NDUFS4 (1:1,000, abcam ab139178, RRID: AB_2922810), NDUFV1 (1:1,000, Proteintech 11238-1-AP, RRID: AB_2149040), NDUFA9 (1:1,000, Thermo Fisher 459100, RRID: AB_10376187), NDUFB11 (1:1,000, Abcam ab183716, RRID: AB_2927481), HTRA2 (1:1,000, R&D AF1458-SP, RRID: AB_2280094), VDAC (1:1,000, Abcam ab14734 RRID: AB_443084), Actin (1:1,000, CST 4967S, RRID: AB_330288), OPA1 (1:1,000, BD 612606, RRID: AB_399888, Lot# 8135704) and IMMT (1:5,000, Proteintech, 10179-1-AP, RRID: AB_2127193). The secondary antibodies used were sheep anti-mouse HRP (1:5,000, Cytiva NA931, RRID: AB_772210) and donkey anti-rabbit HRP (1:5,000, Cytiva, NA934, RRID: AB_772206).

### BN-PAGE westerns

Fifty million K562 cells were resuspended in mitochondrial isolation buffer (0.32 M sucrose, 1 mM EDTA, 10 mM Tris–HCl, pH 7.4) with 1× cOmplete protease inhibitor (Roche) on ice and homogenized with ten strokes of a glass dounce homogenizer. Lysates were centrifuged at 1,000*g* to pellet nuclei and the supernatant was re-centrifuged at 13,000*g* to enrich mitochondria. Pellets were stored at –80 °C until BN-PAGE processing. BN-PAGE was adapted from published protocols^53^. Mitochondria were resuspended at 10 µg µl^−1^ in native PAGE sample buffer and solubilized with 4 µg digitonin (Sigma) per microgram mitochondria for 10 min on ice, followed by centrifugation at 16,000*g* for 30 min at 4 °C. Supernatant was mixed with Coomassie Blue G loading buffer (1:3 volume) and supercomplexes resolved on native PAGE 3–12% Bis–Tris gradient gels (Thermo Fisher Scientific) using dark blue cathode buffer (150 V, 30 min), then light blue cathode buffer (300 V, 90 min). Proteins were transferred to methanol-activated PVDF membranes at 100 V for 60 min and blocked with 5% non-fat milk in TBST before immunoblotting.

### C1 activity assay

Permeabilized-cell respirometry was performed on a Seahorse XFe96 Analyser at 37 °C. Control and *HTRA2* KO K562 cells were seeded at 125,000 cells per well in Cell-Tak-coated (20 µg per well; Corning, 354240) XFe96 microplates and attached by centrifugation (500g, no brake/acceleration). Before the assay, the medium was replaced with MAS buffer (70 mM sucrose, 220 mM mannitol, 10 mM KH_2_PO_4_, 5 mM MgCl_2_, 2 mM HEPES and 1 mM EGTA, pH 7.2) containing 3 nM XF Plasma Membrane Permeabilizer (Agilent, 102504-100), 4 mM ADP (Thermo Fisher, J60672) and C1 substrates (10 mM pyruvate/1 mM malate or 10 mM glutamate/10 mM malate). C1-driven respiration was calculated by subtracting oxygen consumption resistant to piericidin A (Enzo, ALX-380-235-M002) and antimycin A (Sigma, A8674).

### Sedimentation assay

For striatum tissue, mitochondria were isolated from dissected striata by homogenizing frozen hemi-brains in MHSE (210 mM mannitol–5 mM HEPES–70 mM sucrose–1 mM EGTA) buffer (30 strokes, chilled Dounce homogenizer). Homogenates were centrifuged at 600*g* (15 min, 4 °C) to remove debris, and mitochondria were pelleted at 12,000*g* (15 min, 4 °C), washed once in MHSE and resuspended in lysis buffer (30 mM Tris–HCl pH 7.4, 200 mM KCl, 0.5% Triton X-100, 5 mM EDTA, 0.5 mM PMSF and protease inhibitors). Protein concentration was determined and pellets were frozen. For sedimentation, mitochondria (400 µg) were lysed in chilled lysis buffer with protease inhibitors (1,400 rpm, 15 min, 4 °C).

For cell samples, cells were washed in DPBS, pelleted (300*g*, 5 min, 4 °C), and resuspended in hypotonic buffer (20 mM HEPES pH 7.4, 2 mM MgCl_2_, 10 mM KCl, 1 mM DTT and protease inhibitors) at 5× packed cell volume. After 5 min on ice, cells were homogenized through a 25 G needle (6 passes) and cleared twice at 1,000*g* (1 min, 4 °C). The supernatant was layered onto ice-cold 8% glycerol and centrifuged at 10,000*g* (1 min, 4 °C) to pellet mitochondria. Pellets were resuspended in RIPA with protease inhibitors and quantified by the BCA assay.

For the sedimentation assay, equal amounts of lysates were transferred into ultracentrifuge tubes, after balancing the weight with lysis buffer they were spun at 125,000*g* for 20 min at 4 °C. Post-centrifugation, supernatants were collected and precipitated with cold TCA. Both the supernatant and pellet fractions were resuspended in 3× Laemmli buffer for SDS–PAGE/western blot analysis as described above. The primary antibodies used were HTRA2 (1:1,000, Proteintech 15775-1-AP, RRID: AB_2122835, Lot# 00104148), CLPB (1:1,000, Proteintech, 15743-1-AP, RRID: AB_2847900, Lot# 00089384), HAX1 (1:1,000, Proteintech, 11266-1-AP, RRID: AB_2263720, Lot# 00023547), NDUFA13 (1:1,000, Proteintech, 10986-1-AP, RRID: AB_2150609) and TIMM50 (1:1,000, Proteintech, 22229-1-AP, RRID: AB_2879039, Lot# 00124369).

### NAD^+^/NADH measurement

Intracellular NAD^+^ and NADH were measured using the NAD/NADH-Glo Assay (Promega, G9071) following the manufacturer’s instructions. Cells were plated at 4 × 10^5^ cells ml^−1^ in 6-well plates and treated with or without 500 nM rotenone for ~1.5–2 h before collection. Cells were lysed in 1% DTAB, and lysates were split for selective NAD^+^ (acid-treated, 0.4 N HCl) and NADH (base-treated) quantification. Samples were incubated at 60 °C for 15 min, neutralized, combined with detection reagent in white 96-well plates and luminescence recorded after 30–60 min. Values were background-subtracted and normalized to calculate NAD^+^, NADH and NAD^+^/NADH ratios.

### NDI overexpression assay

Control (dummy), *HTRA2* (*HT*) KO and *NDUFS2* (*S2*) KO cells were retrovirally transduced to overexpress yeast Ndi1 (NADH dehydrogenase) or empty vector control. Cells were plated in complete DMEM (10% FBS + penicillin–streptomycin–glutamine) on day 0 in six-well plates and counted using a Beckman Coulter Counter, on day 3 to assess proliferation.

### RNA-seq library preparation and analysis

Total RNA was isolated from ~4-week-old mouse striata and purified using the Direct-zol RNA MicroPrep Kit (Zymo Research, R2062) as described by the manufacturer. RNA-seq library preparation from 500 ng of total RNA was performed according to the manufacturer’s instructions of the QuantSeq 3′ mRNA-Seq V2 Library Prep Kit (Lexogen, 193.384). Final libraries were quantified using a QubitTM 1X dsDNA HS Assay Kit (Thermo Fisher Scientific, Q33231) and subjected to quality control before sequencing. Libraries were single-end sequenced on NovaSeq X Plus.

FASTQ files were processed to assess quality by determining general sequencing bias, clonality and adaptor sequence contamination. RNA-seq reads were aligned to the mm10 mouse reference genome using STAR (v2.7.10a)^54^. Gene expression levels were calculated using HOMER (v4.11)^55^ by counting all strand specific reads within exons. Only the most abundant transcripts, including multiple alternative variants, were selected for each gene, and the genes with a length smaller than 250 bp were removed. Transcripts per million (TPM) were used to evaluate the correlation among replicates. Differential gene expression was calculated using DESeq2 (v1.46.0)^56^ to assess both biological and technical variability between experiments.

### IP and proteomics

HTRA2 and CLPB IPs (*n* = 3 biological replicates per condition, including IgG and no-antibody controls) were performed for proteomics. ~10 million (×3 for IP) cells were crosslinked with 0.2 mM DSP, quenched with 1 M Tris (pH 7.5) and lysed in 0.5% NP40 IP buffer with protease/phosphatase inhibitors. For HTRA2 IP, antibody (~2.5 μg per 1 mg protein) was pre-conjugated to 75 μl Protein G Dynabeads (Thermo Fisher, 10004D); for CLPB IP, antibody (~7 μg per 250 μg protein) was added directly to lysate overnight at 4 °C before bead capture (75 μl, 1–1.5 h, room temperature). Beads were washed in decreasing detergent concentrations (3× 0.05% NP40, 2× detergent-free). Input and flow-through samples were collected for western blotting to assess IP efficiency. Proteins were eluted via on-bead digestion using a urea-based reduction buffer, followed by alkylation with iodoacetamide, reduction with DTT and overnight digestion with trypsin at 37 °C. A second digestion step with a small amount of trypsin was performed the next morning. Peptides were recovered by combining eluates and wash fractions and samples were stored at –80 °C and sent for proteomic analysis to UC Davis Proteomics Core.

For mass spectrometry, peptides (0.6 µg) were separated on an UltiMate 3000 RSLC system using a PepSep C18 column (150 µm × 8 cm, 1.5 µm; 40 °C, 650 nl min^−1^, 60 min gradient) and analysed on an Orbitrap Exploris 480 in DIA mode (spray voltage 1.8 kV, capillary temperature 275 °C). Full MS scans were acquired at 120,000 resolution (*m*/*z* 350–1,400, AGC 300%, 45 ms), with 34 DIA isolation windows (21 Da, NCE 30%, AGC 1,000%). Data were processed in Spectronaut v20 (DirectDIA) as described above for HEK proteomics.

For westerns, beads were dissolved in 30 µl 2× sample buffer, boiled at 95 °C for 5 min with shaking, then briefly spun down and the supernatant collected on a DynaMag as the eluent. The primary antibodies used were HTRA2, CLPB (as described above for WB) and IgG (Proteintech, 30000-0-AP, RRID: AB_2819035).

### Protein expression and purification

PARL-cleaved CLPB was purified as previously described^33^. N-terminal 6xHis-HTRA2 and C-terminal 6xHis-NDUFA13 were expressed in *E. coli* BL21 (DE3) RIL cells (induced with 1 mM IPTG, 15 °C, 16 h). Cells were lysed by high-pressure homogenization in HTRA2 binding buffer (50 mM HEPES–KOH pH 8.0, 750 mM NaCl, 10 mM MgCl_2_, 20% glycerol, 2 mM β-mercaptoethanol and 2 µM pepstatin A) or NDUFA13 binding buffer (8 M urea, 20 mM sodium phosphate pH 8.0, 100 mM NaCl and 1% glycerol). Clarified lysates were incubated with Ni–NTA resin (1 h, 4 °C), washed with 20 mM imidazole and eluted with 300 mM imidazole. Proteins were concentrated and purified by size-exclusion chromatography (HiLoad 16/600 Superdex 200) in HTRA2 storage buffer (50 mM HEPES–KOH pH 8.0, 300 mM NaCl, 10 mM MgCl_2_, 20% glycerol and 1 mM DTT) or NDUFA13 storage buffer (8 M urea, 20 mM sodium phosphate pH 8.0, 100 mM NaCl and 5% glycerol). Peak fractions were pooled, concentrated (10 kDa MWCO), aliquoted and stored at −80 °C. NDUFA13 was maintained in 8 M urea to keep it unfolded for downstream disaggregation assays.

### Luciferase disaggregation and reactivation assay

Aggregates of firefly luciferase were prepared using the protocol described earlier^33^. Firefly luciferase aggregates were prepared by incubating luciferase (50 µM; Sigma, L9420) in luciferase reactivation buffer (LRB: 25 mM HEPES–KOH pH 8.0, 150 mM potassium acetate, 10 mM magnesium acetate and 10 mM DTT) with 8 M urea (Fisher, BP169-10) at 30 °C for 30 min, followed by 100-fold dilution into LRB, snap-freezing and storage at −80 °C. For disaggregation assays, CLPB (1 µM) or HTRA2 (1 µM) was incubated with 50 nM aggregated luciferase in LRB supplemented with an ATP regeneration system (5 mM ATP (Sigma, A2383), 1 mM creatine phosphate (Roche, 10621714001) and 0.25 µM creatine kinase (Roche, 10127566001) at 37 °C for 60 min. Luciferase activity was measured in 96-well optical plates on a Tecan Spark reader, and the remaining reactions were analysed by SDS–PAGE and western blotting for HTRA2-mediated degradation.

### NDUFA13 aggregate cleavage assay

NDUFA13 aggregates were prepared by rapid 100-fold dilution of concentrated, unfolded protein into buffer (25 mM HEPES–KOH pH 8.0, 150 mM potassium acetate, 10 mM magnesium acetate and 10 mM DTT), as previously described^33^. Aggregate formation was confirmed by centrifugation (21,130*g*, 20 min, 4 °C), with 93.3% ± 3.3% of NDUFA13 recovered in the pellet by western blot.

For cleavage assays, CLPB (1 µM), HTRA2 (1 µM) or both were incubated with aggregated NDUFA13 in reaction buffer with an ATP regeneration system (5 mM ATP, 1 mM creatine phosphate and 0.25 µM creatine kinase) at 37 °C for 60 min. Reactions were quenched with 1× Laemmli buffer (95 °C, 5 min) and analysed by SDS–PAGE and western blotting.

### Western blotting and detection

Protein samples were resolved by SDS–PAGE (Bio-Rad Criterion gels) until molecular weight standards (50–75 kDa range) were clearly separated. Proteins were transferred to PVDF membranes using 1× transfer buffer containing 20% methanol at 100 V for 1 h at 4 °C. Membranes were blocked in 5% (w/v) skimmed milk in 1× TBS for 1 h at room temperature, followed by incubation with primary antibodies overnight at 4 °C. Primary antibodies included anti-luciferase (1:1,000, Santa Cruz, sc-74548, Lot# K1922) and anti-NDUFA13 (1:1,000, Proteintech, 10986-1-AP, Lot# 17984). Membranes were washed three times with TBST (1× TBS, 0.2% Tween-20) and incubated with a secondary antibody (1:10,000; LI-COR, 926-68071, 926-68070) for 1 h at room temperature. After additional washes, blots were visualized using a LI-COR Odyssey Fc imaging system.

### Cryo-electron microscopy/electron tomography of isolated mitochondria

WT (*n* = 2; P23 female, P25 male) and *Htra2* mutant (*n* = 2; P19, P27 males, near end stage) mice were sacrificed and their brains isolated (excluding olfactory bulb) at 4 °C. Tissue was minced in mitochondrial isolation buffer (50 mM Tris–HCl, 250 mM sucrose, 1 mM EDTA, 1% BSA, pH 7.4), Dounce homogenized (30 strokes) and centrifuged at 600*g* (10 min). Supernatants were pooled and mitochondria pelleted at 12,000*g* (15 min), resuspended in buffer with 200 nM MitoTracker Green and kept on ice before vitrification^31,57^.

Glow-discharged gold 100-mesh grids were loaded with 5 µl sample, sandwiched between hexadecene-coated planchettes and vitrified by high-pressure freezing (CryoCapCell). Lamellae (~100–180 nm) were prepared on a Helios 5 Hydra plasma focused ion beam scanning electron microscope using the Waffle method^58^. Cryo-electron tomography data were collected on a 300 kV Titan Krios G4 with tilt series from −60° to +60° (3° increments, 64,000×, 1.96 Å per pixel, ~123 e⁻ Å^−^^2^). Tilt series were CTF-corrected, aligned and reconstructed in Warp (v2.0.0dev36)/AreTomo (v1.4.0); single images were processed in cryoSPARC (v4.7.0). Mitochondrial dimensions were measured in Fiji (v2.14.0/1.54 f) from 15,000× SearchMaps.

### Statistics and reproducibility

Sample sizes were based on related prior datasets^2^. Sample sizes were greater than three, except for K562 proteomics data collection, where *n* = 2 for controls, which represents a limitation of our work with respect to in vitro proteomics data. One biological replicate from both the CLPB IP and striatum proteomics datasets was excluded owing to insufficient peptide counts. Statistical tests used to generate *P* values are indicated in the respective figure legends. Data distribution was assumed to be normal throughout all experiments, although this was not formally tested. All biochemical experiments were performed with at least three replicates, and key experiments were independently repeated by a separate laboratory member. Animals were randomly allocated for all in vivo experiments. Investigators were blinded during immunofluorescence scoring analysis.

### Reporting summary

Further information on research design is available in the Nature Portfolio Reporting Summary linked to this article.

## Supplementary information

Supplementary Information Supplementary Figs. 1–7.

Reporting Summary

Supplementary Data 1 Statistical source data.

## Source data

Source Data Fig. 1 Unprocessed brain IF images.

Source Data Fig. 1 Statistical source data.

Source Data Fig. 2 Unprocessed western blots.

Source Data Fig. 2 Statistical source data.

Source Data Fig. 3 Unprocessed western blots.

Source Data Fig. 3 Statistical source data.

Source Data Fig. 4 Unprocessed western blots.

Source Data Fig. 4 Statistical source data.

Source Data Extended Data Fig. 1 Statistical source data.

Source Data Extended Data Fig. 2 Statistical source data.

## Data Availability

HEK proteomics and mouse striatum proteomics data are deposited in MassIVE under accession code MSV000101385. IP proteomics and K562 proteomics data are deposited in MassIVE under accession code MSV000101384. All RNA-seq data are deposited in GEO under accession number GSE327486. Source data are provided with this paper.

## Code availability

No custom code was used for this study.
