# Supplementary material for: Hypoxia rescues complex 1-associated disease caused by proteostatic defects
Source: Nat Metab. 2026 Jul 8;8(8):1791–804. doi: 10.1038/s42255-026-01566-0 (PMC13489956; doi:10.1038/s42255-026-01566-0)
Supplement: Supplementary file 1 [file 42255_2026_1566_MOESM10_ESM.pdf]

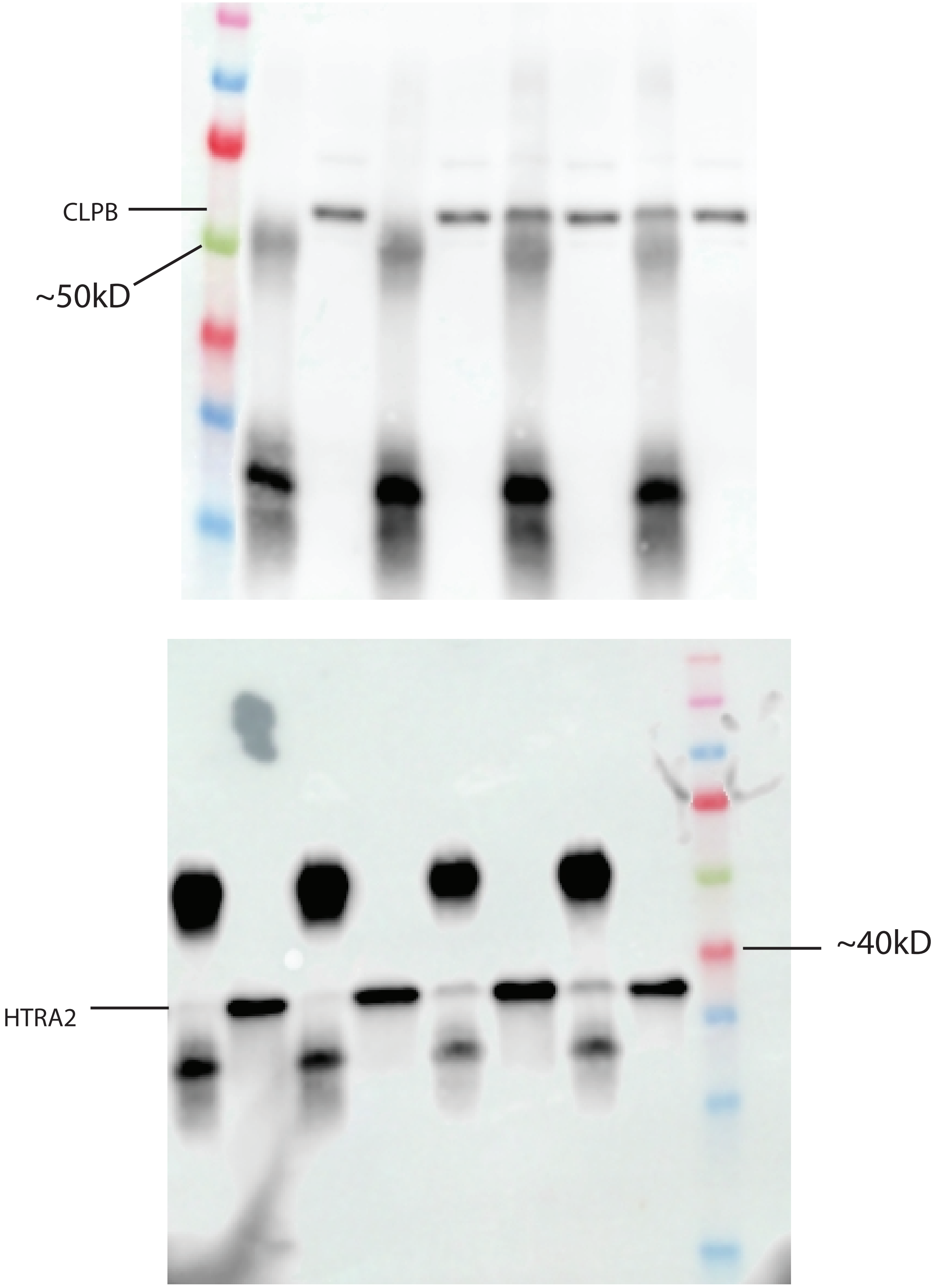

Fig 4B

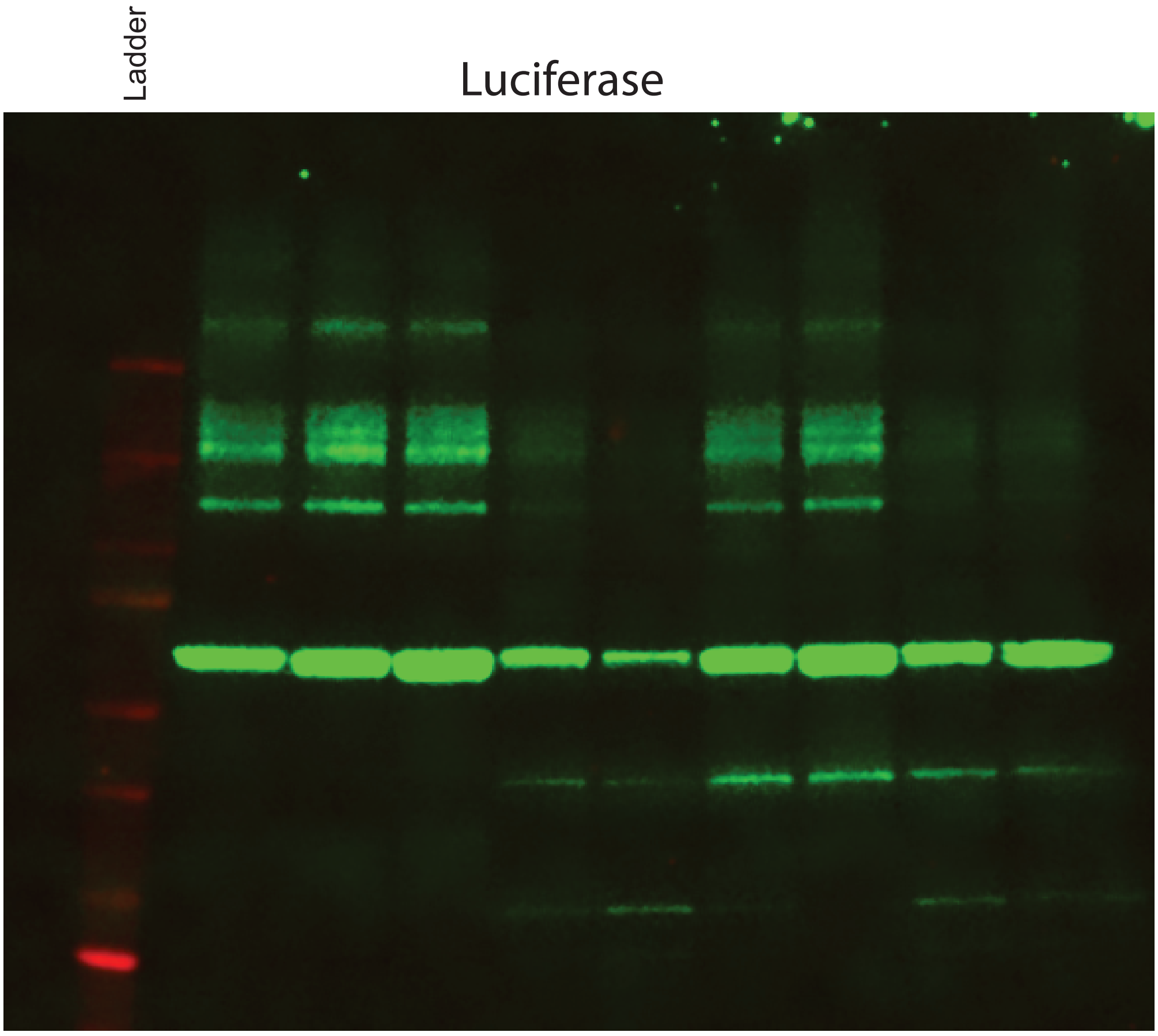

Fig 4C

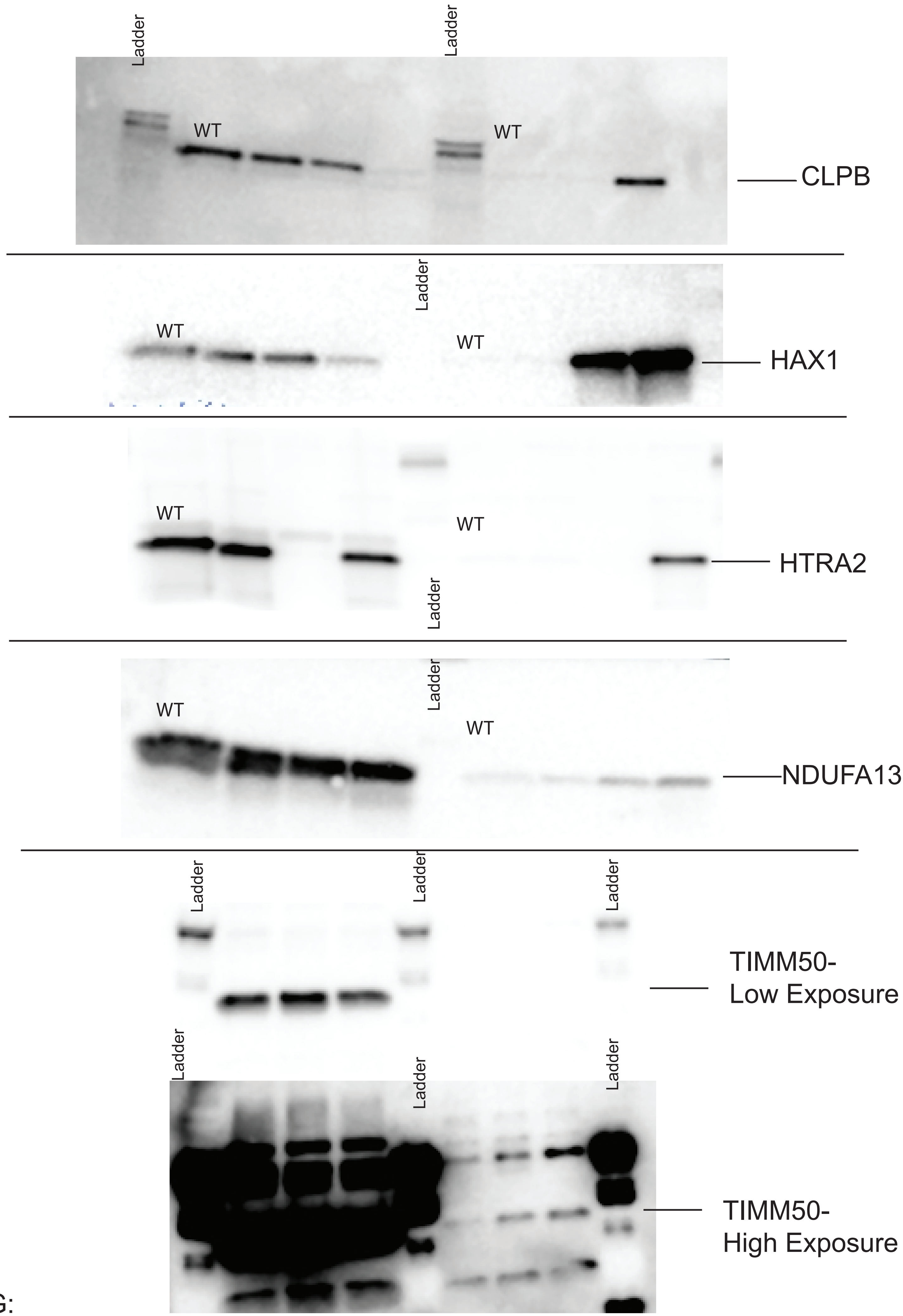

Fig 4G:
WT data is not shown in main figure
(Non-targeting/dummy guideRNAs are in figure)

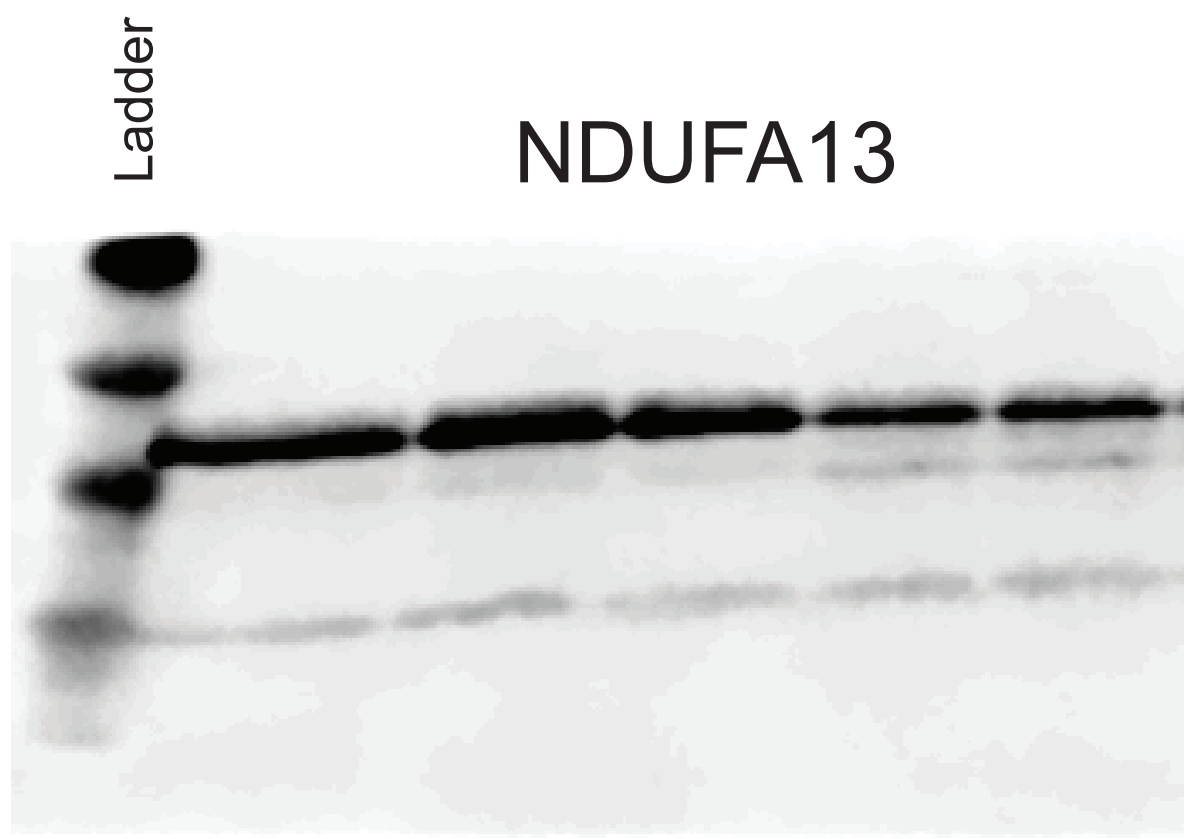

Fig 4I
