# Supplementary material for: Hypoxia rescues complex 1-associated disease caused by proteostatic defects
Source: Nat Metab. 2026 Jul 8;8(8):1791–804. doi: 10.1038/s42255-026-01566-0 (PMC13489956; doi:10.1038/s42255-026-01566-0)
Supplement: Supplementary file 2 [file 42255_2026_1566_MOESM1_ESM.pdf]

nature metabolism

Supplementary information https://doi.org/10.1038/s42255-026-01566-0

Hypoxia rescues complex 1-associated disease caused by proteostatic defects

In the format provided by the authors and unedited

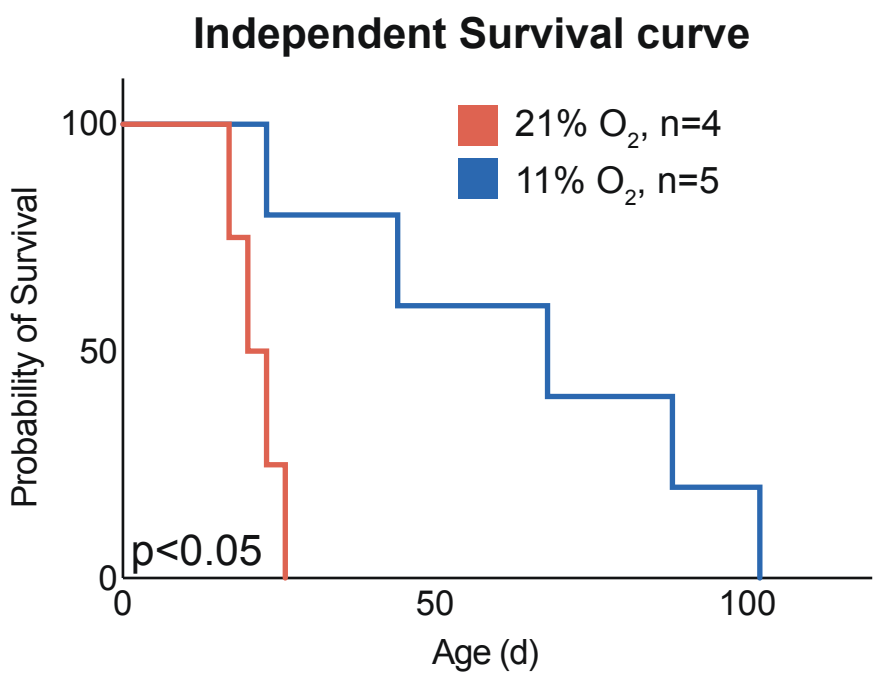

Fig. S1. Independent Validation of Hypoxia Lifespan Extension of Htra2 Mutant Mice in Second Cohort. Kaplan–Meier survival curve of Htra2 mutant mice exposed to hypoxia in an independent survival cohort. n = 4 for normoxia (21% O_2); n = 5 for hypoxia (11% O_2). p-value was calculated using the Log-rank (Mantel–Cox) test; $p < 0.05$.

NAD⁺/NADH is unaffected in *HTRA2* KO cells

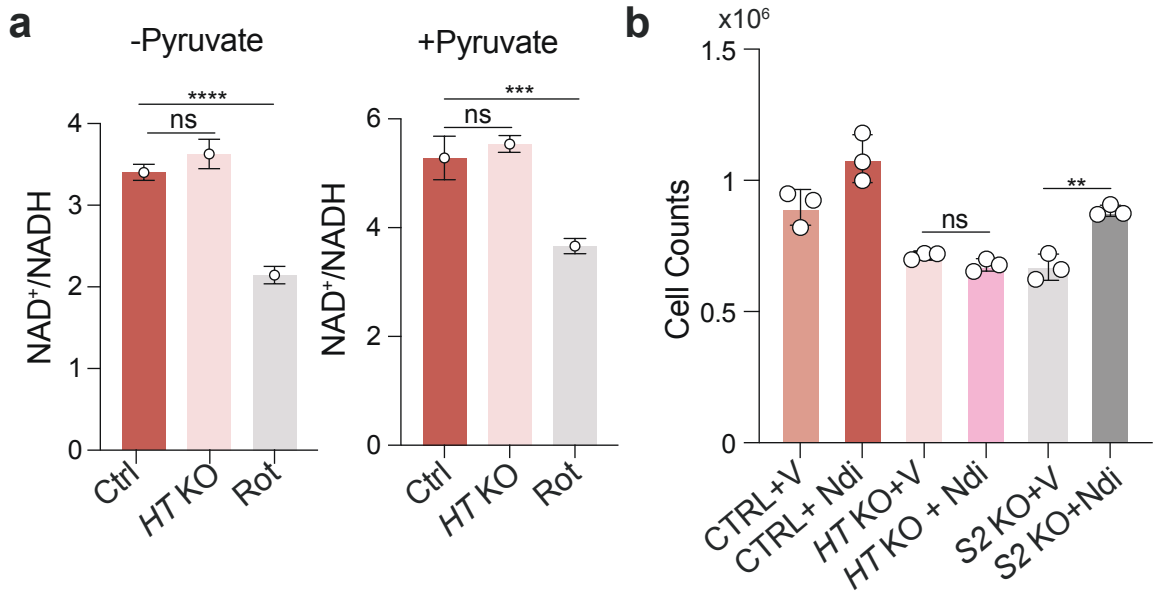

Fig. S2. NAD /NADH levels are unchanged in HTRA2 KO cells in vitro. (a) Intracellular NAD and NADH were quantified using the NAD/NADH-Glo™ luminescent assay in control, HTRA2 (HT) KO, and rotenone-treated control (Rot) cells. Luminescence values were background-subtracted to calculate NAD , NADH, and NADH/NAD ratios. Data are representative of two biological replicates and are presented as mean ± SEM. (b) Control, HTRA2 (HT) KO, and NDUFS2 (S2) KO cells were retrovirally transduced to overexpress yeast Ndi1 (NADH dehydrogenase) or empty vector control. Cells were plated on day 0 in 6-well plates and counted on day 3 to assess proliferation. Data are representative of 3 biological replicates and are presented as mean ± SD. Statistical significance was determined using unpaired two-tailed t-test; **p< 0.005.

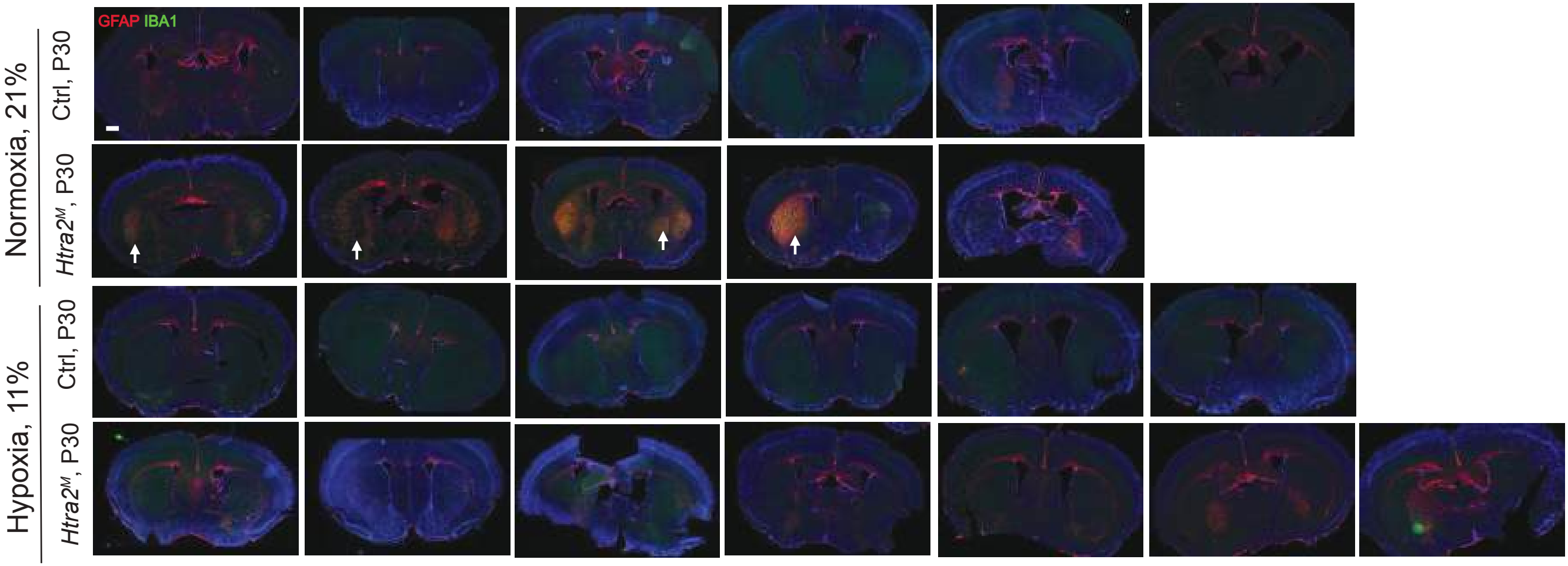

Fig. S3. Full Neuropathology Set for Striatum. Additional neuropathology replicates (including samples shown in Fig. 1), used for blinded scoring of lesions in the striatum (indicated by white arrows) in Htra2 mutant mice under normoxic and hypoxic conditions. Iba1 (green), Gfap (red), and DAPI (blue) staining were performed on brains of control and mutant mice exposed to hypoxia starting at P11. Brains were harvested at ~P30 for all groups. Representative images are shown from multiple animals per group. Scale bar- 300µm

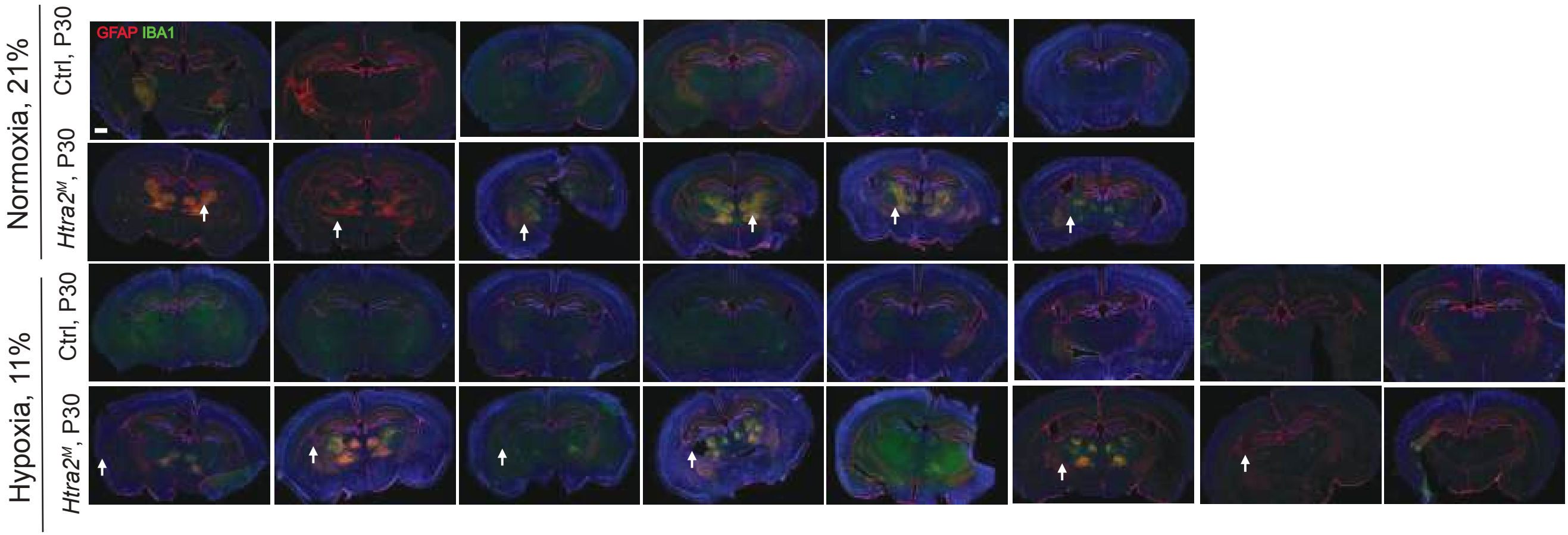

Fig. S4. Full Neuropathology Set for Thalamus. Additional neuropathology replicates (including samples shown in Fig. 1), used for blinded scoring of lesions in the thalamus (indicated by white arrows) in Htra2 mutant mice under normoxic and hypoxic conditions. Iba1 (green), Gfap (red), and DAPI (blue) staining were performed on brains of control and mutant mice exposed to hypoxia starting at P11. Brains were harvested at ~P30 for all groups. Representative images are shown from multiple animals per group. Scale bar- 300µm

**Pathways negatively enriched in KO**	**padj**
Respiratory electron transport	1.90 E-12
Aerobic respiration and respiratory electron transport	2.95 E-11
Complex I biogenesis	3.59 E-07
Mitochondrial translation initiation	0.03
Mitochondrial translation elongation	0.03
Mitochondrial translation termination	0.03

Fig. S5. Pathway enrichment analysis of proteomics data from HTRA2 knockout K562 cells. Pathway enrichment analysis was performed using g:Profiler. Reactome pathways are shown for proteins that were downregulated in HTRA2 knockout cells, using a cutoff of fold change ≤ 0.9 and $p < 0.05$ (Student's t-test, two-tailed, equal variance), suggested that mitochondrial Complex I biogenesis and aerobic respiration are downregulated, consistent with our observation that Complex I subunits are decreased in HTRA2 knockout cells.

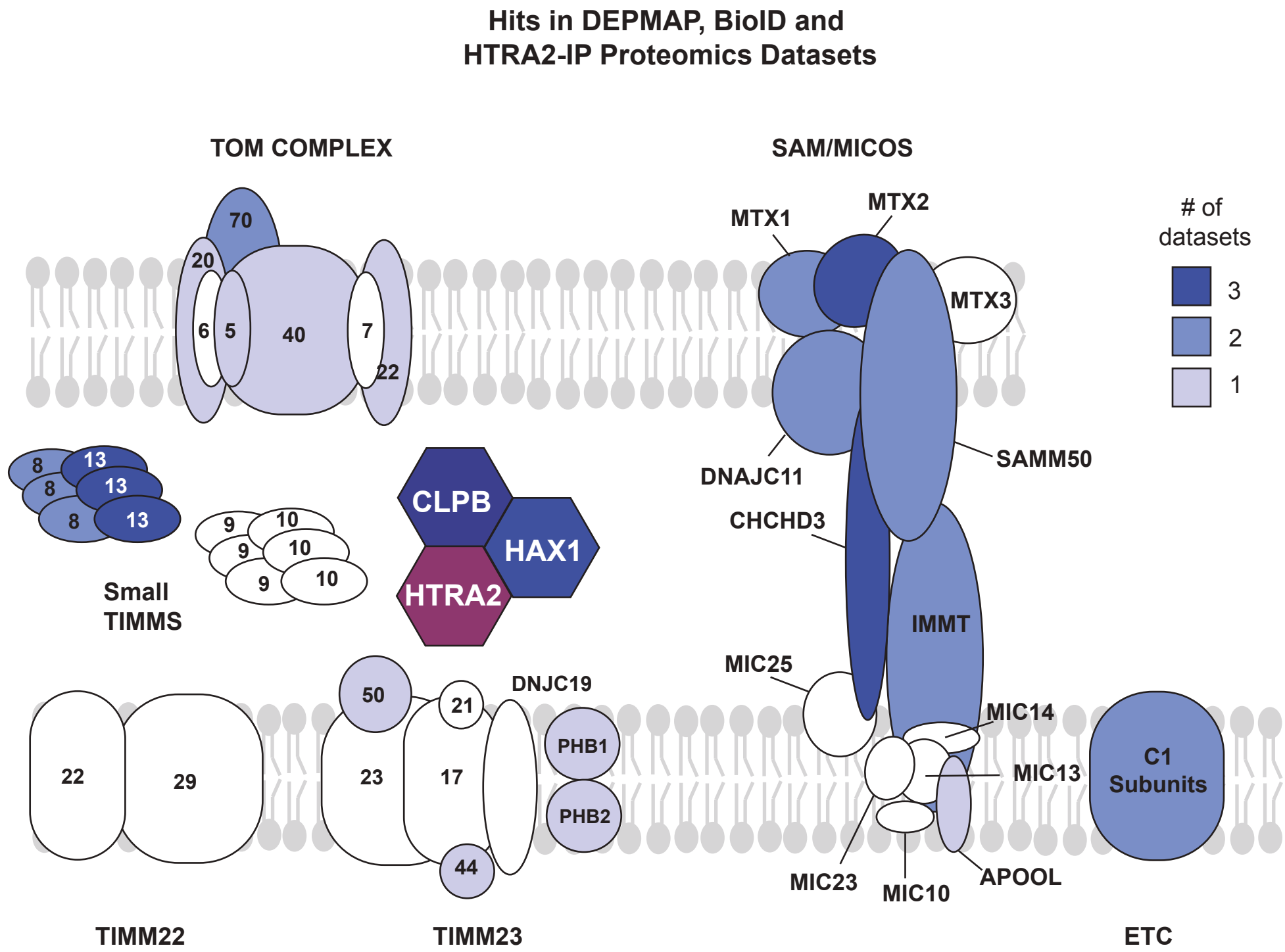

Fig. S6. Relationships Between Key Mitochondrial Intermembrane Space Proteins Using Published Datasets. The DepMap dataset, BioID proximity labeling with HTRA2, and HTRA2 immunoprecipitation (IP) proteomics were further analyzed independently to identify proteins that are co-essential with and interact with HTRA2. The DepMap and BioID datasets were generated by other research groups (as described in Fig. 3). Integrating these datasets revealed that the HTRA2 complex (with CLPB and HAX1) plays a role in regulating the transport of proteins through the TIM/TOM complexes, MICOS assembly, and the function of prohibitins—ultimately impacting mitochondrial cristae structure and the assembly of ETC subunits involved in oxidative phosphorylation. The shaded blue color indicates the presence of a protein across multiple datasets; the darker the shade, the more datasets in which the protein was identified as a hit.

Mitochonrial Cristae are abnormal In *Htra2^M* brain

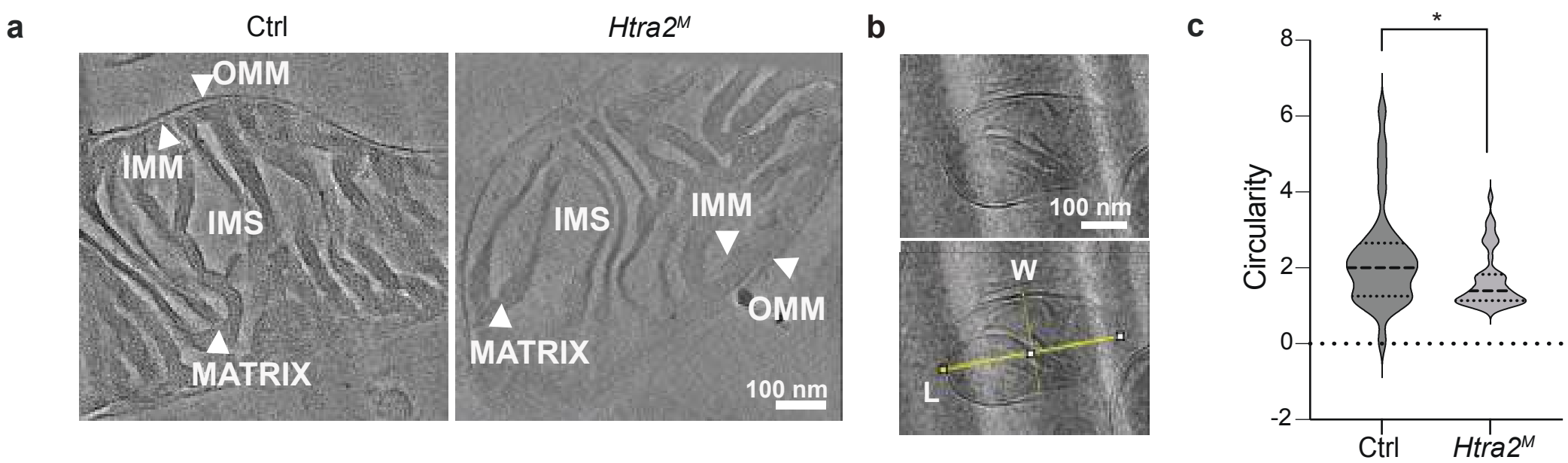

Fig. S7. Cryo-EM/ET shows Htra2 mutant has abnormal mitochondrial cristae structure and increased circularity. (a) TEM images of control (wild-type) isolated mouse brain mitochondria (left) and tomogram reconstruction of Htra2 mutant mitochondria (right) at 64000x. (b-c) TEM image of control mitochondria at 15000x, and representative image showing the length (L) and width (W) measurement used to calculate circularity (C=L/W) of control and mutant mitochondria. Mitochondria from control (n=30) and mutant (n=42) brains were analyzed. Data are presented as violin plot to visualize the distribution. An unpaired two-tailed t-test was performed. *$p < 0.05$
