# Supplementary material for: Hypoxia rescues complex 1-associated disease caused by proteostatic defects
Source: Nat Metab. 2026 Jul 8;8(8):1791–804. doi: 10.1038/s42255-026-01566-0 (PMC13489956; doi:10.1038/s42255-026-01566-0)
Supplement: Supplementary file 4 [file 42255_2026_1566_MOESM4_ESM.pdf]

Fig1H-Striatum-HET-206-21pc

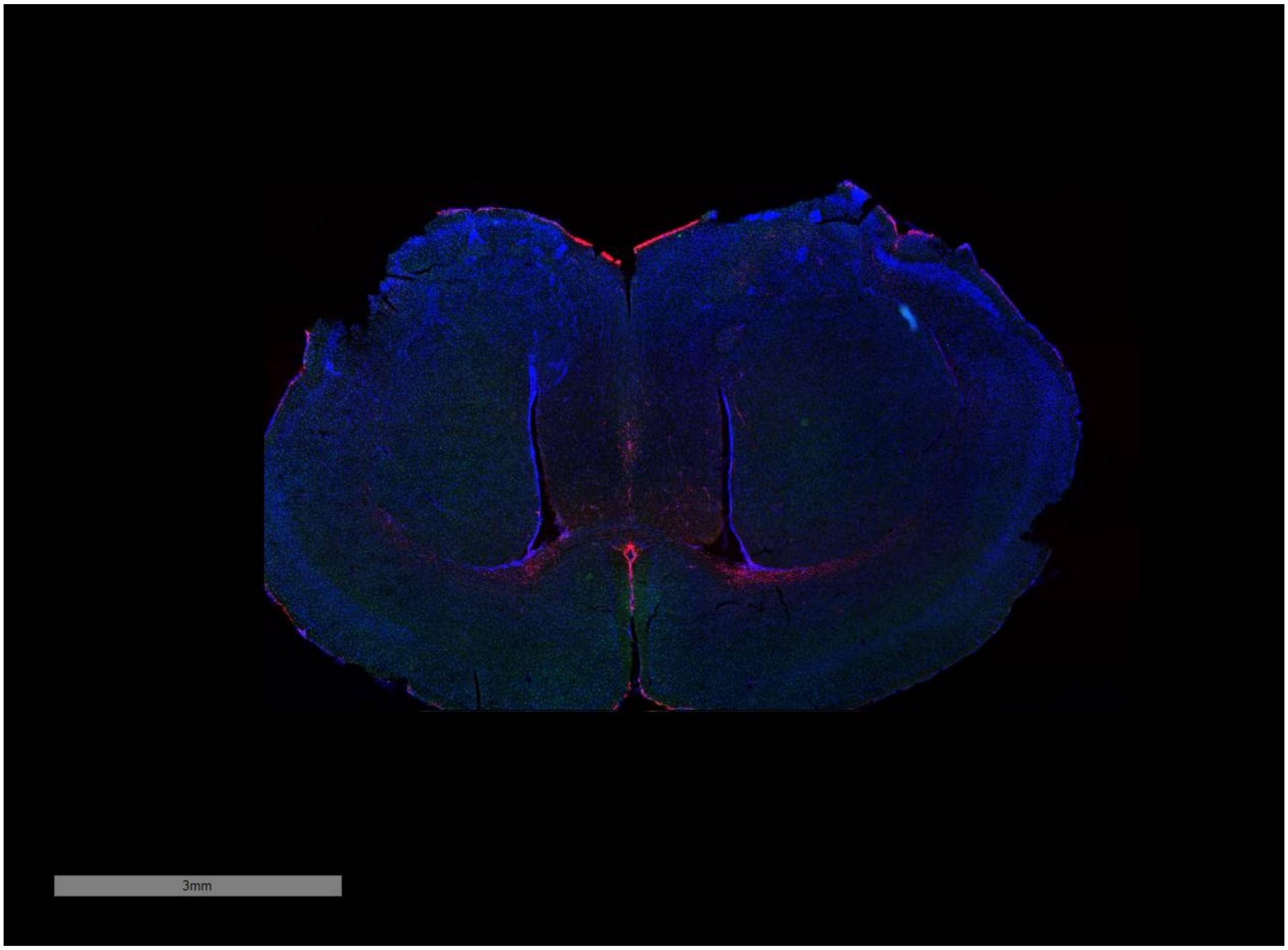

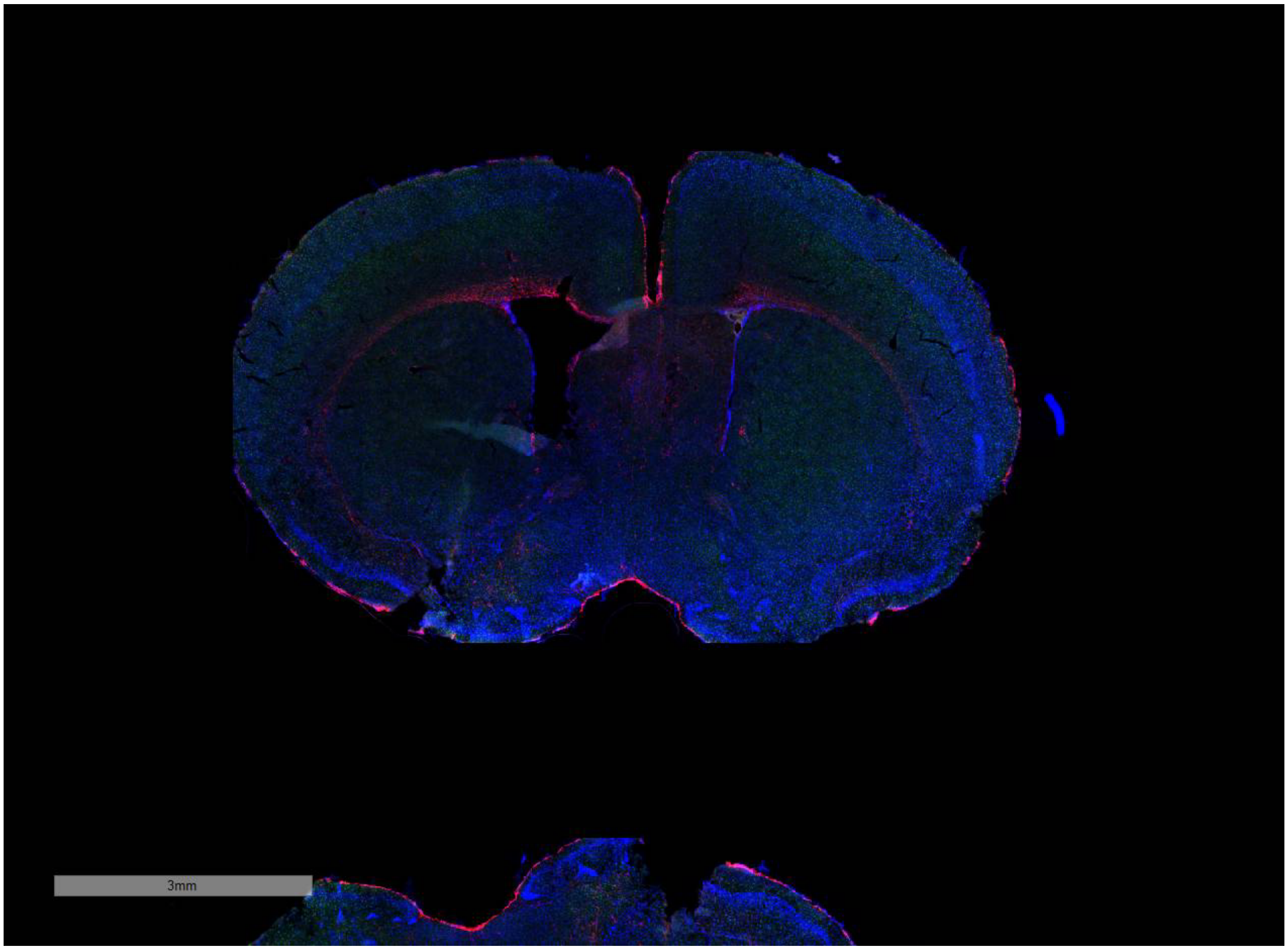

Fig1H-Striatum-HET-G1-11pc

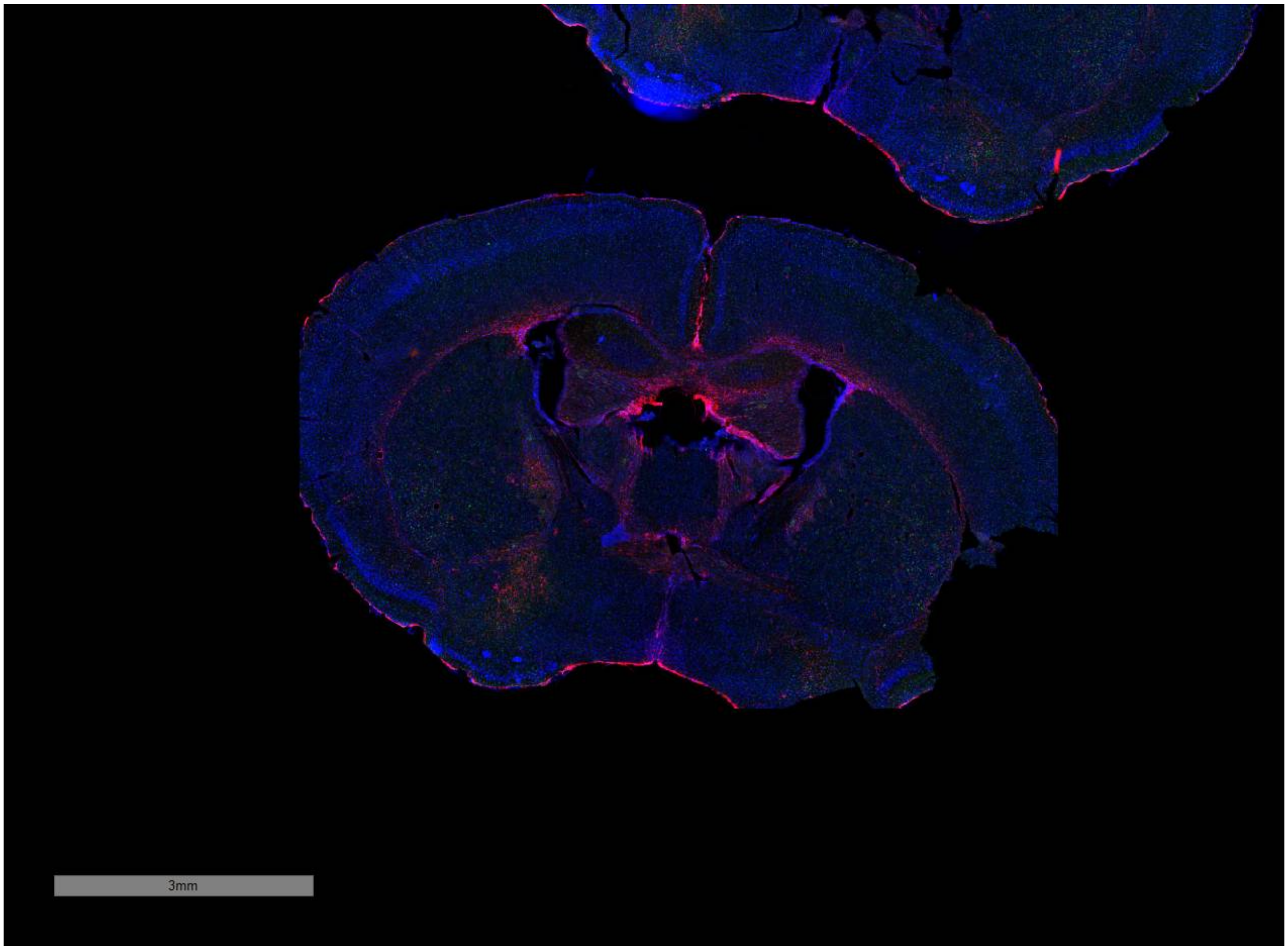

Fig1H-Striatum-Mut-221-11pc

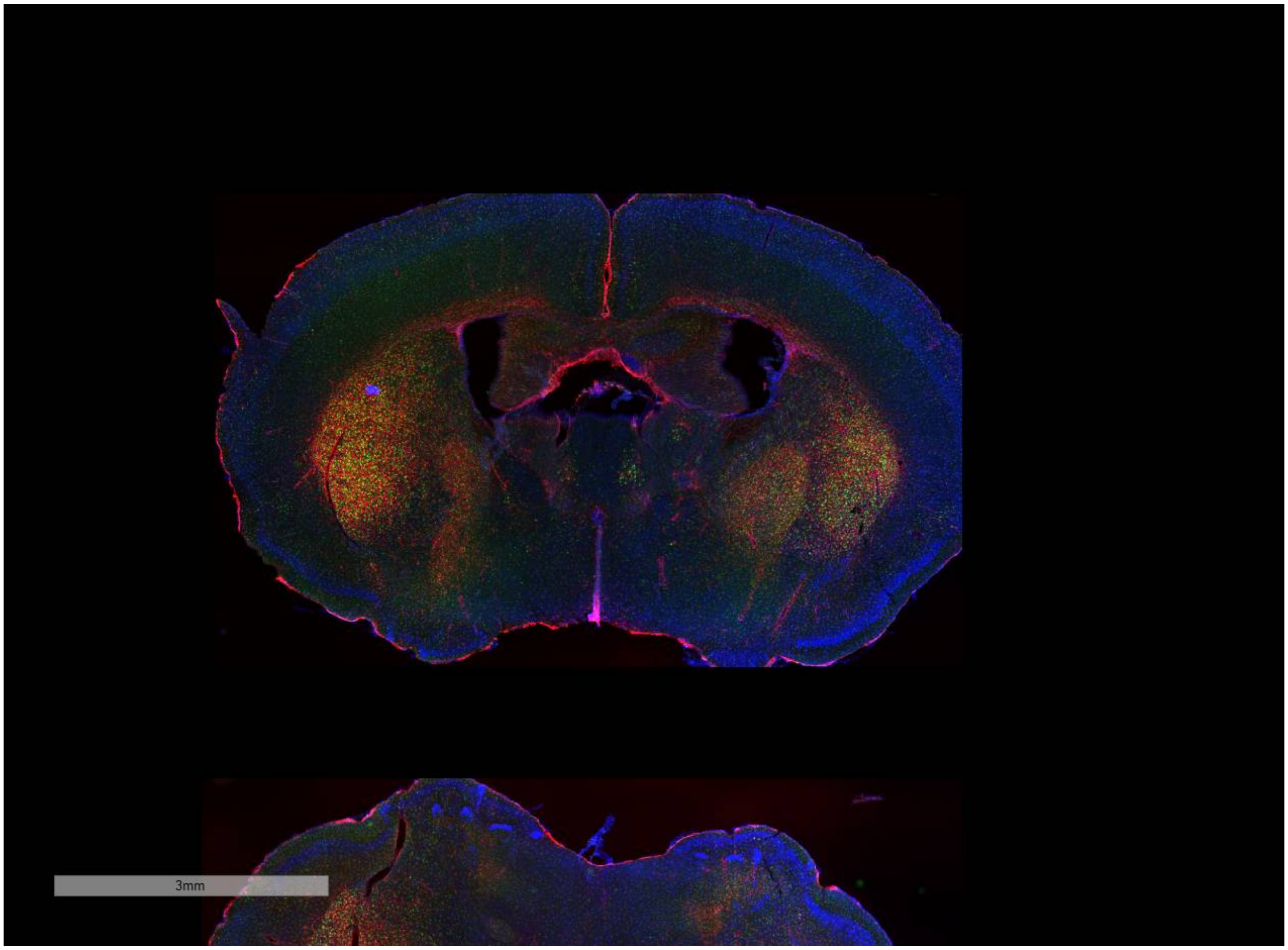

Fig1H-Striatum-Mut-B-21pc

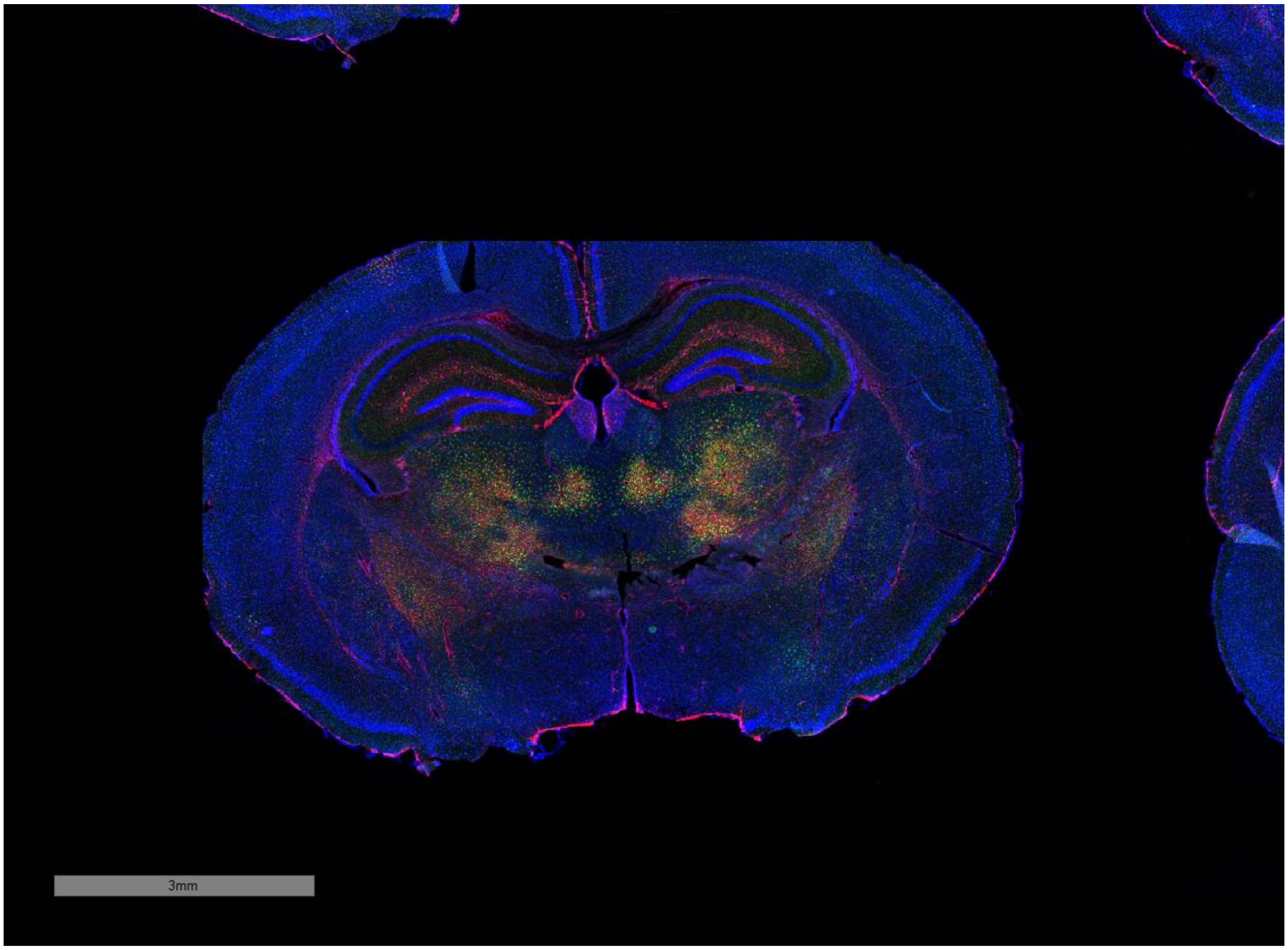

Fig1H-Thalamis-Mut-D1-11pc

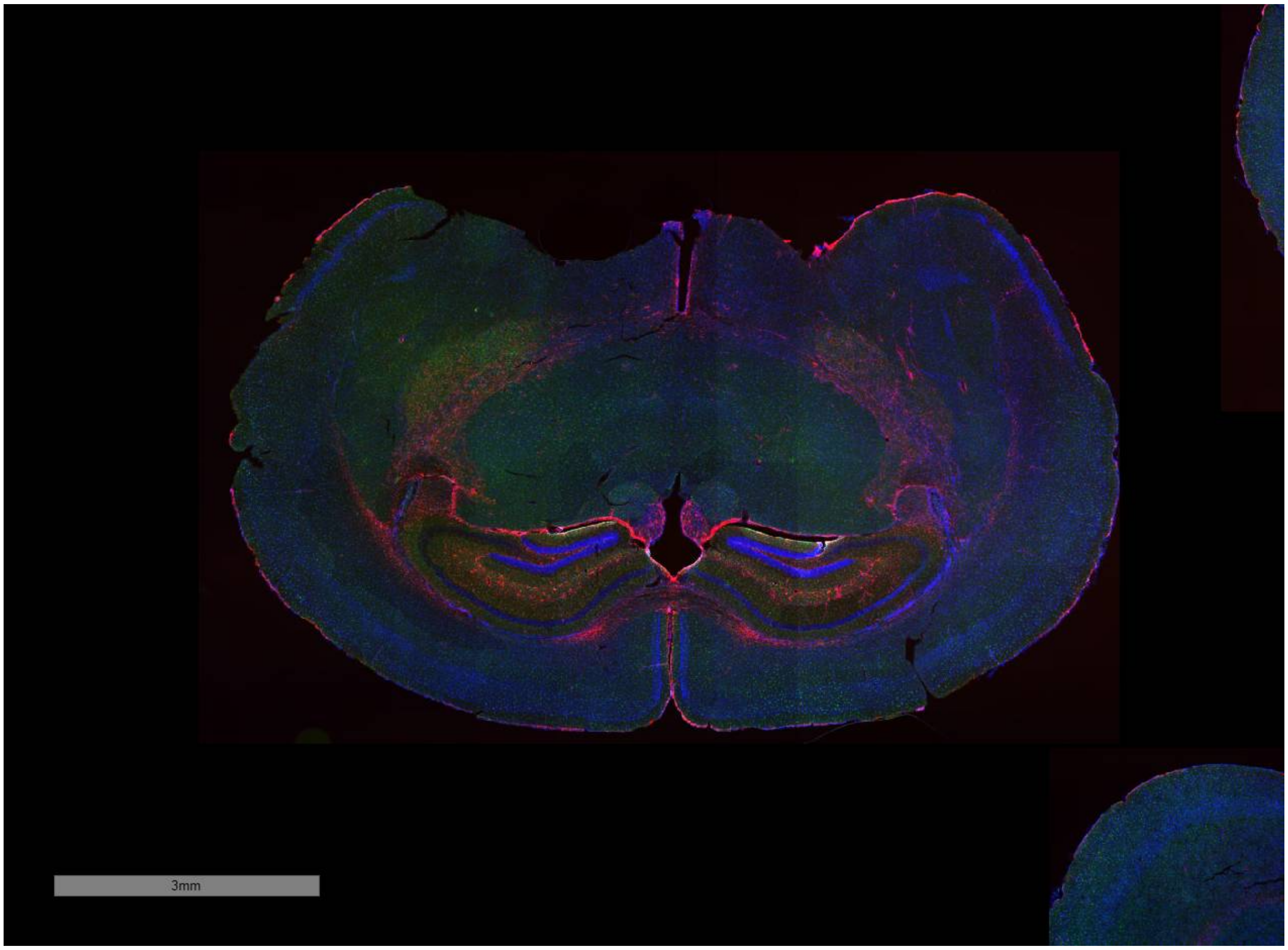

Fig1H-Thalamus-HET-208-21pc

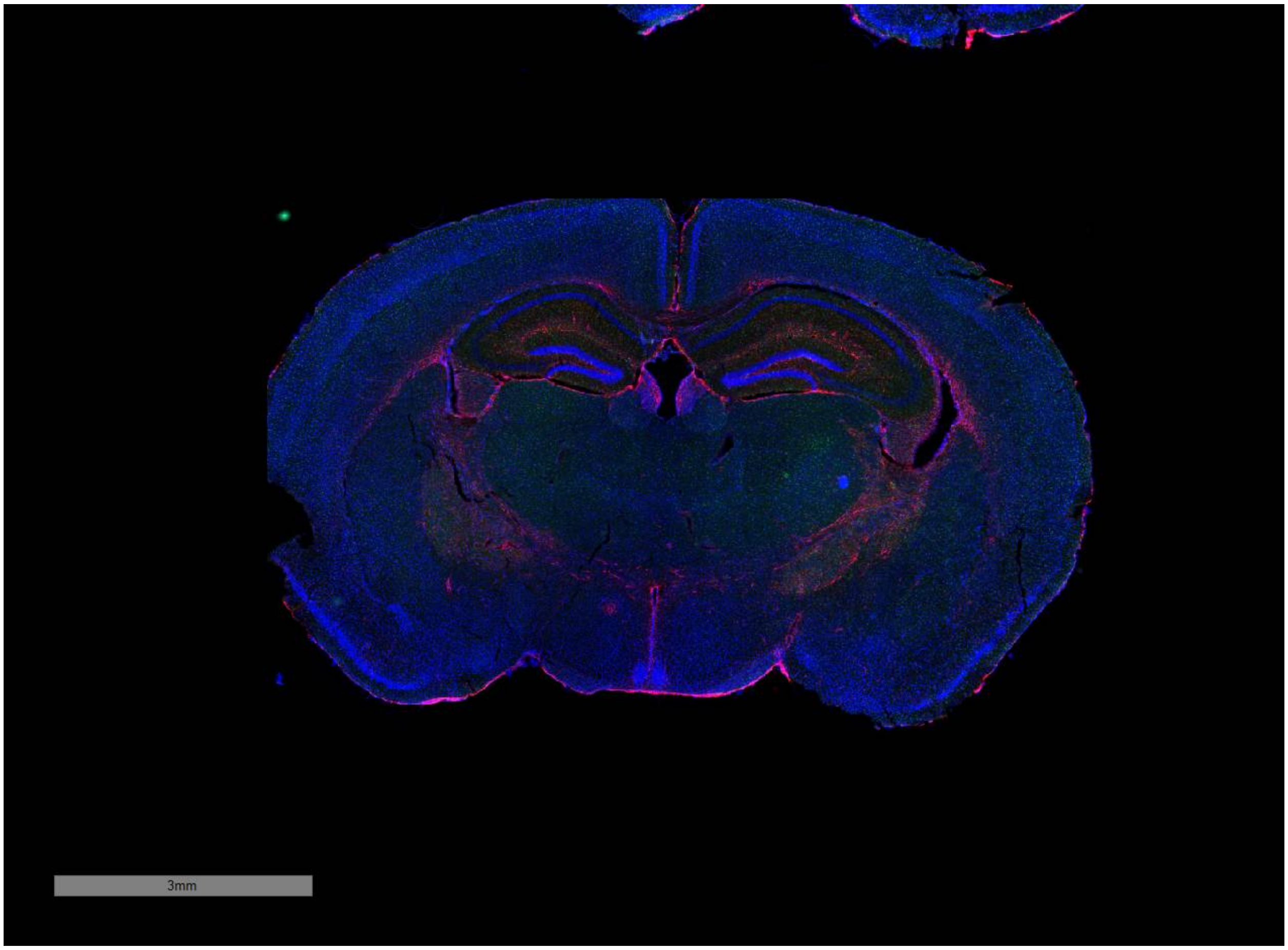

Fig1H-Thalamus-HET-G1-11pc-0.9x-5

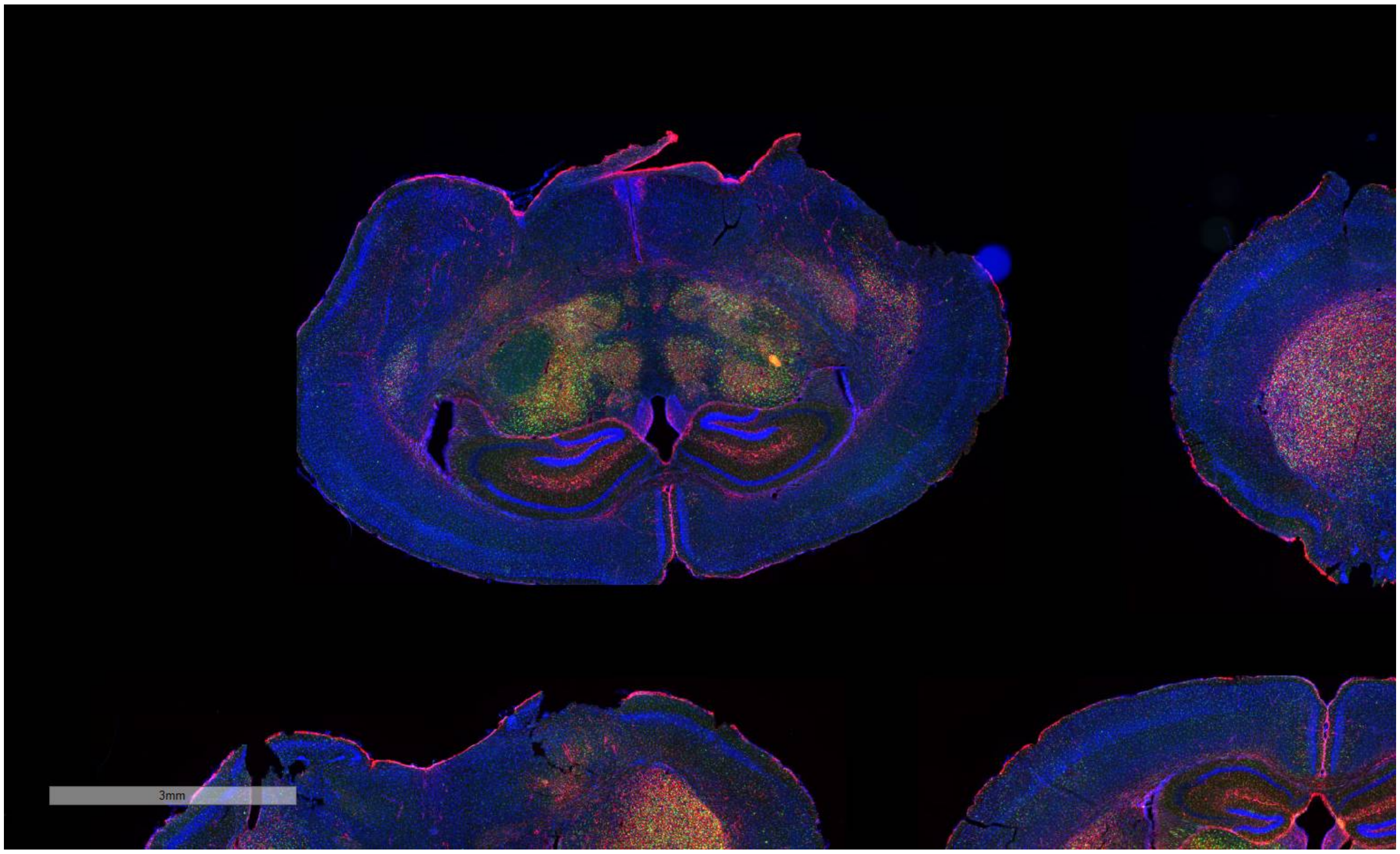

Fig1H-Thalamus-Mut-C-21pc
