# Supplementary material for: Hypoxia rescues complex 1-associated disease caused by proteostatic defects
Source: Nat Metab. 2026 Jul 8;8(8):1791–804. doi: 10.1038/s42255-026-01566-0 (PMC13489956; doi:10.1038/s42255-026-01566-0)
Supplement: Supplementary file 5 [file 42255_2026_1566_MOESM6_ESM.pdf]

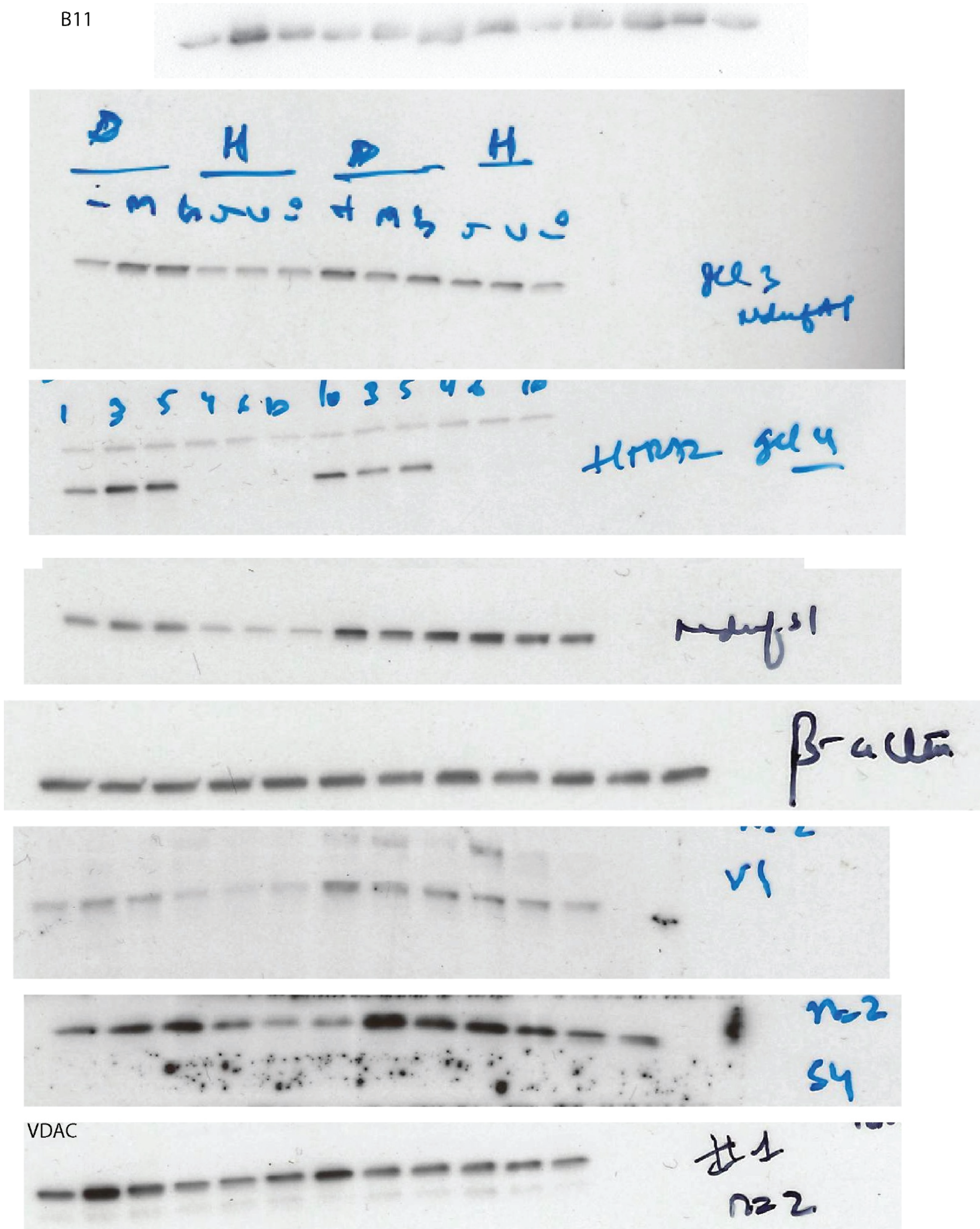


Fig 2D

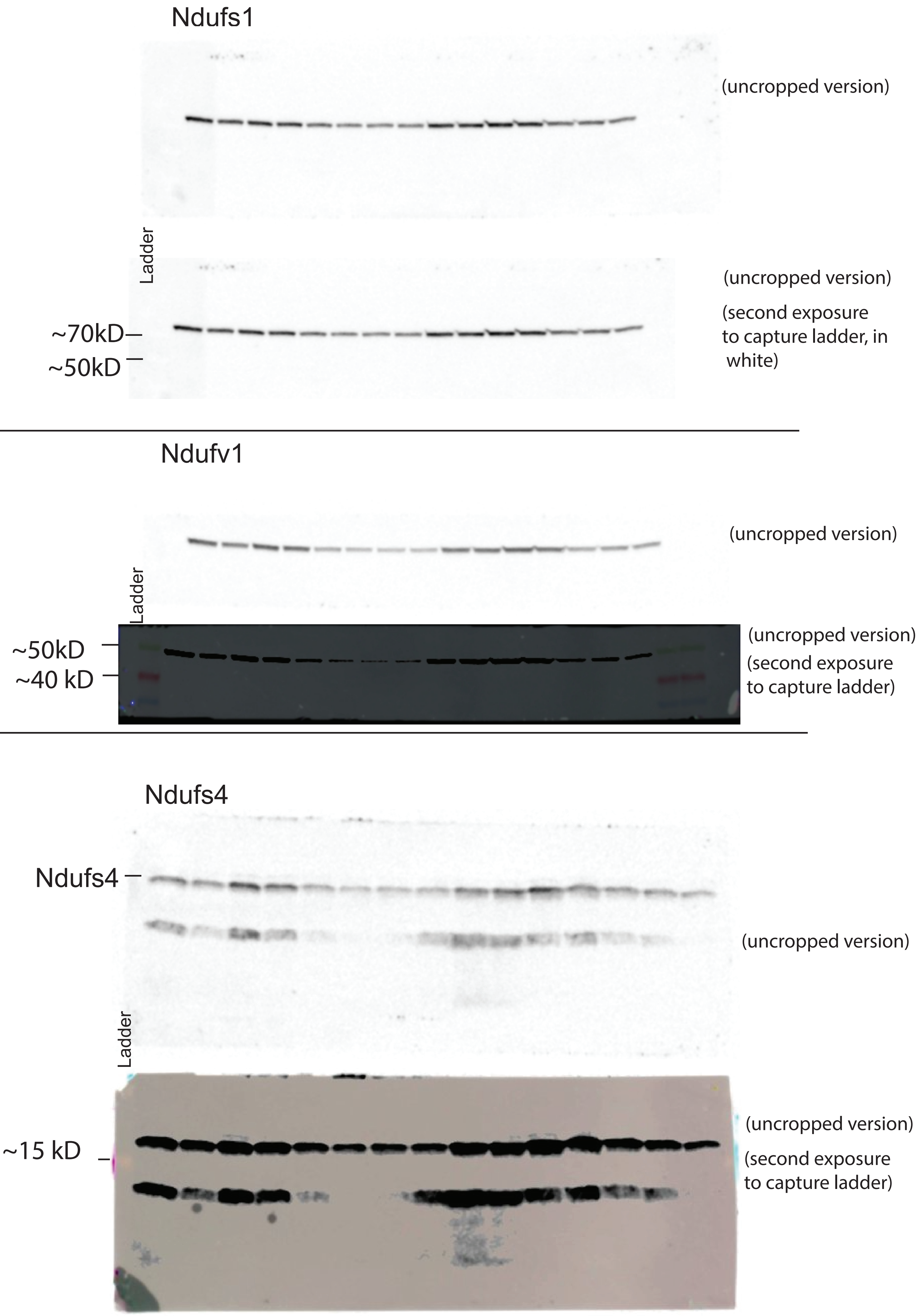
Ndufs1
(uncropped version)
Ladder
~70kD
~50kD
(uncropped version)
(second exposure
to capture ladder, in
white)
Ndufv1
(uncropped version)
Ladder
~50kD
~40 kD
(uncropped version)
(second exposure
to capture ladder)
Ndufs4
Ndufs4
(uncropped version)
Ladder
~15 kD
(uncropped version)
(second exposure
to capture ladder)

Fig 2H

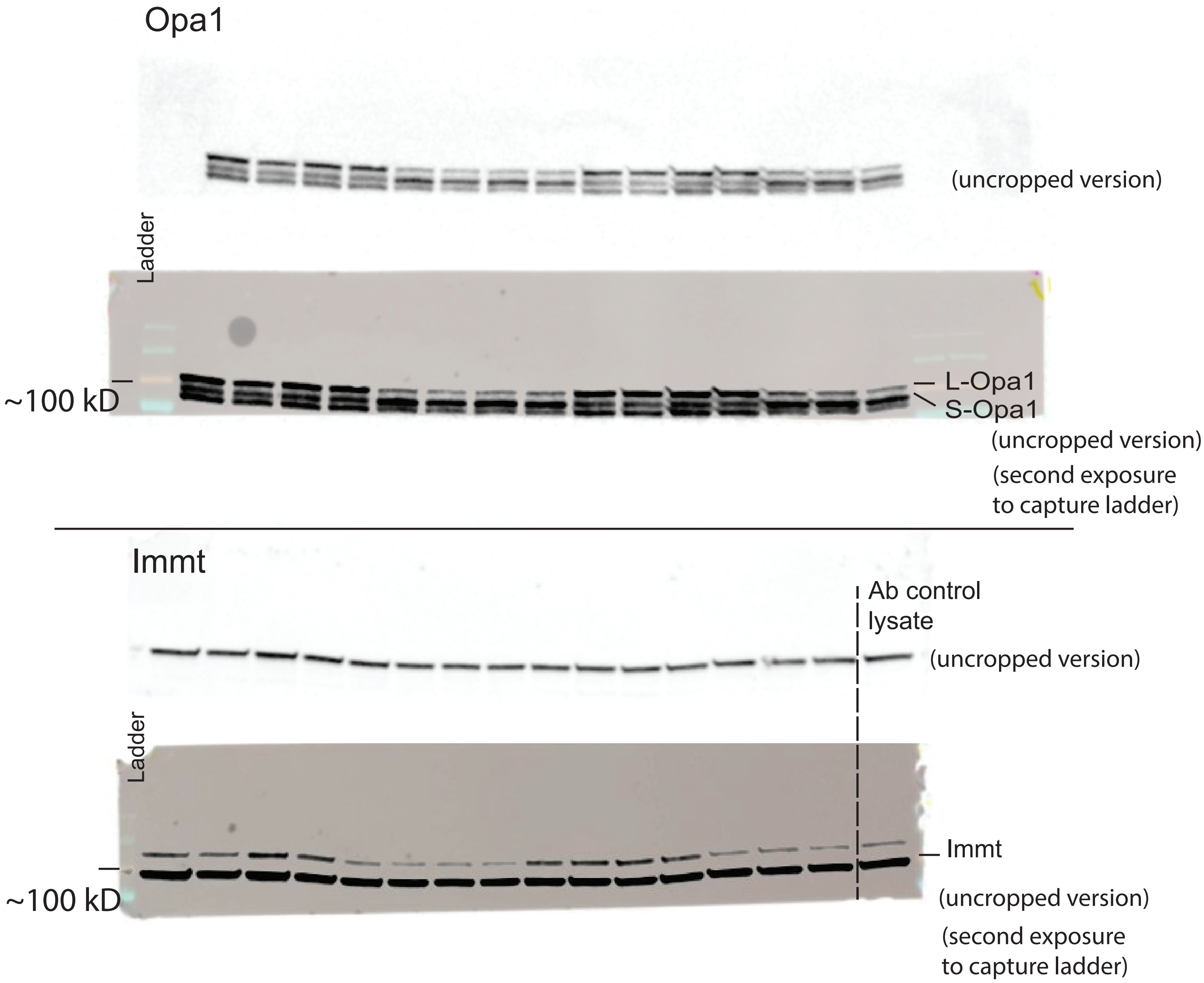
Opa1
(uncropped version)
Ladder
~100 kD
L-Opa1
S-Opa1
(uncropped version)
(second exposure
to capture ladder)
Immt
Ab control
lysate
(uncropped version)
Ladder
~100 kD
Immt
(uncropped version)
(second exposure
to capture ladder)

Fig 2H
